# Transposon Dysregulation Modulates dWnt4 Signaling to Control Germline Stem Cell Differentiation in *Drosophila*

**DOI:** 10.1371/journal.pgen.1005918

**Published:** 2016-03-28

**Authors:** Maitreyi Upadhyay, Yesenia Martino Cortez, SiuWah Wong-Deyrup, Leticia Tavares, Sean Schowalter, Pooja Flora, Corinne Hill, Mohamad Ali Nasrallah, Sridar Chittur, Prashanth Rangan

**Affiliations:** 1 Department of Biological Sciences/RNA Institute, University at Albany SUNY, Albany, New York, United States of America; 2 NYU Langone Medical Center, New York, New York, United States of America; 3 Boston University School of Medicine, Boston, Massachusetts, United States of America; 4 Department of Environmental Health Sciences, University of Massachusetts Amherst, Amherst, Massachusetts, United States of America; 5 CFG Core Facility, University at Albany SUNY, Rensselaer, New York, United States of America; Johns Hopkins University School of Medicine, UNITED STATES

## Abstract

Germline stem cell (GSC) self-renewal and differentiation are required for the sustained production of gametes. GSC differentiation in *Drosophila* oogenesis requires expression of the histone methyltransferase dSETDB1 by the somatic niche, however its function in this process is unknown. Here, we show that *dSETDB1* is required for the expression of a Wnt ligand, *Drosophila* Wingless type mouse mammary virus integration site number 4 (dWnt4) in the somatic niche. dWnt4 signaling acts on the somatic niche cells to facilitate their encapsulation of the GSC daughter, which serves as a differentiation cue. dSETDB1 is known to repress transposable elements (TEs) to maintain genome integrity. Unexpectedly, we found that independent upregulation of TEs also downregulated *dWnt4*, leading to GSC differentiation defects. This suggests that *dWnt4* expression is sensitive to the presence of TEs. Together our results reveal a chromatin-transposon-Wnt signaling axis that regulates stem cell fate.

## Author Summary

Every multicellular organism is made up of tissues that are maintained by stem cells, due to their capacity to both self-renew and differentiate into terminal cell types. Loss of either of these processes can lead to aging, progression towards degenerative diseases and cancers. Insight into how self-renewal and differentiation are regulated will have tremendous therapeutic impact. *Drosophila* is an excellent model system for stem cell study due to the availability of various mutants, markers and RNAi technology. We study the ovaries of the female *Drosophila*, whose stem cell population gives rise to gametes. The tissue from which these gametes arise is surrounded by somatic cells, which provide signaling cues required for proper self-renewal and differentiation. While the mechanisms by which these somatic signals regulate stem cell self-renewal is known, how somatic cues regulate differentiation remains unclear. Here, we have identified that transposons, selfish genetic elements, modulate *dWnt4* expression in the somatic cells. We demonstrate that *dWnt4* promotes the somatic encapsulation of the stem cell daughter by regulating adherens junction proteins, thereby promoting differentiation. Transposons have been linked to cancers, and therefore establishing how transposons regulate genes essential for differentiation can provide new perspectives on their role in cancer.

## Introduction

**Fig 1:**
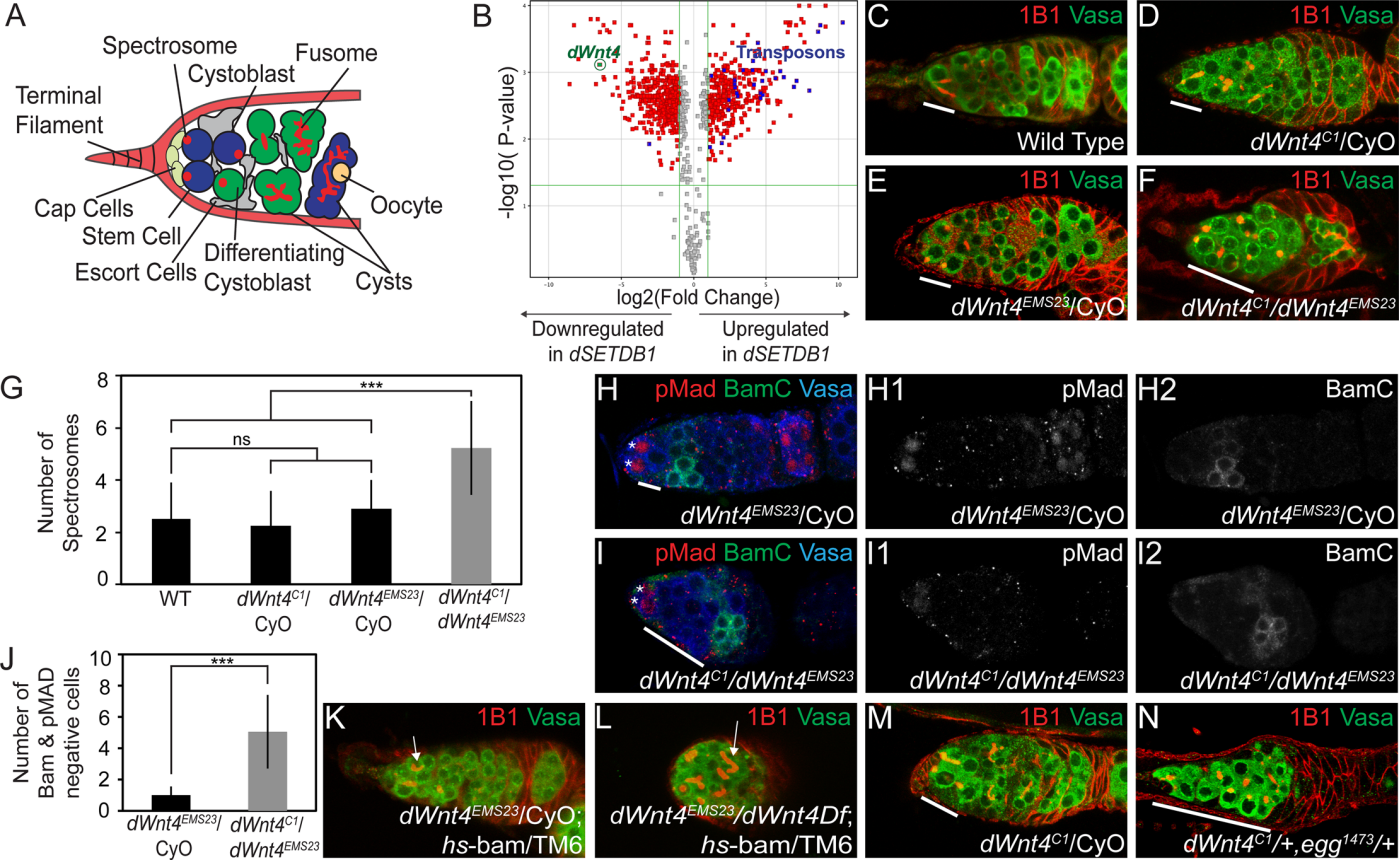
*dWnt4* is required for cystoblast differentiation. (A) A schematic of the *Drosophila* female germarium present at the anterior-most end of each ovariole. Stem cells (blue) are attached to the cap cells (light green). The stem cells divide to give rise to the cystoblast (blue) that turns on expression of Bam, and is referred to as the differentiating cystoblast (green). The cystoblast then undergoes four incomplete mitotic divisions to give rise to a 16-cell cyst (blue). The germline is surrounded by the somatic niche composed of the terminal filament (red), cap cells (light green) and escort cells (grey). (B) Microarray analysis of *dSETDB1* mutants compared to *bam* mutants showing that transposons (blue) are upregulated and *dWnt4* (green) is downregulated. (C-F) Wild type, *dWnt4* heterozygotes and *dWnt4* mutants stained with 1B1 (red) and Vasa (green) showing an accumulation of >3 undifferentiated cells in *dWnt4* mutants (white line). (G) Quantification of the number of single cell spectrosomes in wild type (n = 50), *dWnt4* heterozygotes *dWnt4*^*EMS23*^ (n = 90), *dWnt4*^*C1*^ (n = 40) and *dWnt4* mutants (n = 90) showing a significant difference. (H-I2) *dWnt4* heterozygote and *dWnt4* mutant stained with pMad (red) (white asterisk), BamC (green), and Vasa (blue) showing that *dWnt4* mutants accumulate pMad and Bam negative cells (white line). pMad is shown in H1 and I1, BamC is shown in H2 and I2. (J) Quantification of number of pMad and Bam negative cells in *dWnt4* heterozygote (n = 22) and *dWnt4* mutants (n = 24) showing a significant increase in *dWnt4* mutants. (K-L) *dWnt4* heterozygote and *dWnt4* mutants carrying a *hs-*bam transgene stained with 1B1 (red) and Vasa (green) showing differentiating cysts marked by fusomes (red) post heat shock (white arrow). (M-N) *dWnt4* heterozygote and *dWnt4*, *dSETDB1* trans-heterozygote stained with 1B1 (red) and Vasa (green) showing an accumulation of >3 undifferentiated single cells (white line) in the trans-heterozygote.

*Drosophila* female germline stem cells (GSCs) are an excellent tractable model system to study the mechanisms that regulate stem cell division and differentiation [1–3]. GSCs reside at the anterior end of the ovaries in a structure called the germarium. GSCs divide to give rise to a stem cell daughter or a cystoblast (CB). The CB then turns on a differentiation factor, *bag of marbles* (*bam*) that is both necessary and sufficient to drive differentiation into a sixteen-cell cyst (Fig 1A) [4,5]. One of these sixteen cells becomes an oocyte, while the other fifteen cells become nurse cells.

Both intrinsic and extrinsic factors regulate GSC self-renewal and differentiation into an oocyte [1,2,6]. Two extrinsic factors regulating GSC self-renewal are structural support and Decapentaplegic (Dpp) signaling provided by the terminal filament, cap cells and escort cells located proximally in the somatic niche (Fig 1A) [6,7]. Within the niche, the terminal filament and cap cells provide signaling for GSC self-renewal, while the escort cells physically enclose CBs, allowing for their proper differentiation (Fig 1A) [8,9]. However, signaling pathways that regulate escort cell encapsulation, thereby promoting GSC differentiation, have not been fully elucidated.

dSETDB1 (also known as Eggless [Egg]), a histone methyltransferase, trimethylates histone 3 lysine 9 (H3K9me3) to initiate heterochromatin formation [10]. It activates the transcription of piwi interacting RNAs (piRNAs), which are critical for controlling transposable elements (TEs) to protect genome integrity [11,12]. These piRNAs, with their bound Argonaute proteins, such as Piwi, Aubergine (Aub) and Argonaute 3 (Ago3), target TEs for transcriptional and post-transcriptional silencing [13–15]. *dSETDB1* is also an extrinsic factor required in the escort cells to promote GSC differentiation through an undetermined mechanism [11]. Intriguingly, like the loss of *dSETDB1*, somatic loss of *piwi* or mutations in the somatic piRNA clusters, such as *flamenco*, also results in GSC differentiation defects [11,16,17]. It was proposed that the loss of GSC differentiation observed in *piwi* mutants is due to Dpp over expression [18]. However, it was recently demonstrated that although Dpp upregulation in *piwi* mutants contributes to GSC differentiation, it is not one of the major controlling factors [16]. Therefore, we hypothesized that up-regulation of TEs in somatic cells could modulate an as yet unidentified signaling cue that promotes GSC differentiation.

*Wnt* signaling is critical for maintaining various stem cell systems [19]. Wnts are secreted lipid-modified proteins that mostly act over short distances [19]. Secreted Wnts bind to receptors such as Frizzled 2 (Fz2), activating downstream signaling [20]. The binding of Wnt to these receptors results in stabilization of a downstream effector called β-catenin (*Armadillo* in *Drosophila*) [20]. β-catenin then translocates to the nucleus, where it binds to the TCF/LEF family of transcription factors, activating transcription of downstream targets [20]. There are two Wnt ligands that have been shown to play an important role in *Drosophila* oogenesis, *wingless* (*wg*) and *dWnt4*. *wg* acts later in the differentiation process to regulate follicle stem cells that envelop the developing cysts [21]. *dWnt4* function is required for successful somatic cell migration in the developing gonad [22]. *dWnt4* mutants exhibit ovariole ensheathment defects and have been shown to be female sterile [22].

In line with our hypothesis, we have identified the Wnt ligand dWnt4, downstream of *dSETDB1*, to control GSC differentiation. We find that *dWnt4* acts in an autocrine manner in the escort cells of the stem cell niche to promote somatic encapsulation of CB. We have identified the gap junction protein Innexin 2 (*inx2*), and proteins of the adherens junction (AJ) complex as downstream targets of this dWnt4 signaling. These AJ proteins regulate the encapsulation of the CB by the escort cells. Additionally, piRNA pathway mutations that specifically upregulate TEs in the soma also downregulate *dWnt4* expression and lose CB encapsulation, thereby causing differentiation defects. This suggests that the presence of TEs modulates dWnt4 signaling and somatic encapsulation, thereby affecting GSC differentiation.

## Results

### *dWnt4* acts downstream of *dSETDB1* to promote CB differentiation

To test the hypothesis that a signaling pathway from the soma controls GSC differentiation, and to identify candidate genes involved in signaling from the niche, we performed microarray analysis comparing ovaries from *dSETDB1* mutants with those from *bam* mutants. Both mutants are arrested at the same developmental stage, resulting in the accumulation of CBs [10,11,23]. While *bam* mutants contain heterochromatic marks in both the somatic cells and the germ line, *dSETDB1* mutants lack heterochromatic marks in either cell type [11]. As predicted by our hypothesis, we found that transposons were upregulated (Fig 1B). Intriguingly, we also observed that the *Drosophila* *W**ingless type mouse mammary virus i**nt**egration site number* *4* (*dWnt4*) was strongly downregulated, indicating a potential inhibition of the Wnt signaling pathway (Fig 1B and S1A Fig).

dWnt4 is a conserved Wnt ligand critical for *Drosophila* oogenesis [22]. In mice, Wnts have been shown to mediate encapsulation of the germ line by the surrounding somatic cells, although upstream regulators have yet to be identified [24]. If *dSETDB1* controls GSC differentiation through *dWnt4*, then loss of *dWnt4* should result in GSC differentiation defects. To test this, we analyzed two markers of differentiation, Vasa and 1B1. Vasa stains all germ cells, while 1B1 stains the endoplasmic reticulum-rich organelles known as spectrosomes in the undifferentiated GSCs and CBs, and the branched structures called the fusomes in differentiating cysts (Fig 1A) [25,26]. *dWnt4* mutants accumulated undifferentiated cells, identified by their number of spectrosomes (Fig 1C–1G) (Table 1). In addition, we found pleiotropic differentiation defects in later stages that included fused egg chambers and oocyte specification defects (S1B–S1E Fig). Thus, *dWnt4* is essential for normal GSC differentiation.

**Table 1:** Quantitation of undifferentiated cells in mutants compared to control germaria.

Genotype	Spectrosomes	n	P-value
Wild type	2.5 ± 1.4	50	
*dWnt4*^*EMS23*^/CyO	2.9 ± 1.1	90	
*dWnt4*^*C1*^/CyO	2.2 ± 1.3	40	
*egg*^*1473*^/CyO	2.3 ± 1.3	50	
*Inx2* ^*G0035*^/Fm7c	2.5 ± 1.0	57	
*dWnt4* ^*EMS23*^*/dWnt4*^*C1*^	5.2 ± 1.8	90	2.85381E-20 (compared to *dWnt4*^*EMS23*^/CyO) 2.94448E-16 (compared to *dWnt4*^*C1*^/CyO)
*dWnt*^*C1*^*/egg*^*1473*^	5.4 ± 1.7	52	2.35597E-15 (compared to *dWnt4*^*C1*^/CyO) 1.17107E-16 (compared to *egg*^*1473*^/CyO)
*dWnt4* ^*EMS23*^*/egg*^*1473*^	4.7±1.5	34	5.49755E-11 (compared to *egg*^*1473*^/CyO) 1.64451E-11 (compared to *dWnt4*^*EMS23*^/CyO)
*dWnt4*^*C1*^/*Inx2*^*G0035*^	5.2 ± 2.3	57	3.10871E-10 (compared to *dWnt4*^*C1*^/CyO) 3.59458E-12 (compared to *Inx2*^*G0035*^*/*Fm7c)
*c587*	2.1 ± 1.1	50	
*c587>dWnt4*RNAi	7.7 ± 2.9	56	1.80E-27
*c587>Fz2*RNAi	5.7 ± 1.9	55	6.36033E-21
*c587>DE-Cadherin*RNAi	5.9 ± 2.5	53	1.7529E-14
*c587>β-catenin*RNAi	9.7 ± 8.8	56	2.79694E-08

Mean number of cells containing spectrosomes ± standard deviation for the indicated genotypes determined in Z-stacks by confocal microscopy. P-values for the same were determined by two-tailed equal variance t test by comparing the respective mutants vs. wild type strains.

If *dSETDB1* and *dWnt4* act together, then they should perturb differentiation at the same step. Loss of *dSETDB1* results in the accumulation of undifferentiated CBs, which can be identified by both the diminished expression of the GSC marker phosphorylated Mothers against dpp (pMad), and a lack of expression of the differentiating CB marker, Bam [11,23,27]. Similarly in *dWnt4* mutants, we found an accumulation of cells expressing neither pMad, nor high levels of Bam, suggesting that *dWnt4* mutants also accumulate early CBs (Fig 1H and 1J and S1F and S1G Fig). This result was not due to an increase in the niche size, as indicated by staining for cap cell marker, Lamin C, nor by an increased rate of GSC division, as measured by staining for the mitotic marker, PH3 (17% in *dWnt4* heterozygous germaria, n = 66, compared to 23% in *dWnt4* germaria n = 60, P-value = 0.99) (S1H–S1K Fig) [28]. To determine if these accumulated, undifferentiated CBs were capable of differentiating, we induced the expression of the differentiation factor, Bam, under the control of a heat shock promoter. After heat shock, like in *dSETDB1* mutants [11], we observed the development of cysts, as revealed by the formation of fusomes (80% in *dWnt4* heterozygous germaria were found to form fusomes, n = 40, compared to 85% in *dWnt4* germaria n = 34 P-value = 0.5485) (Fig 1K and 1L) [5]. These results taken together suggest that *dWnt4*, like *dSETDB1*, is required for proper Bam-mediated CB differentiation.

To determine whether *dSETDB1* and *dWnt4* act through the same pathway to promote CB differentiation, we generated flies that were heterozygous for both *dWnt4* and *dSETDB1* and stained for Vasa and 1B1. 60% (n = 77) of germaria accumulated greater than 3 undifferentiated cells in the trans-heterozygote germaria compared to 4% (n = 43) for *dWnt4* heterozygote and 16% (n = 50) in *dSETDB1* heterozygotes, suggesting that *dSETDB1* and *dWnt4* do indeed act through the same pathway to control differentiation (Fig 1M and 1N and S1L and S1M Fig) (Table 1). If *dWnt4* is downstream of *dSETDB1* and heterochromatin formation, then the loss of *dWnt4* should not cause a decrease in *dSETDB1*-dependent heterochromatin formation, nor an increase in the number of double-strand breaks due to TE upregulation [11,29]. Indeed, we observed no differences in heterochromatic marks using H4K20me3, nor increase of TE levels using qRT-PCR, nor increase of double-strand breaks using H2Av staining in *dWnt4* mutants compared to the control (S2A–S2J Fig). Furthermore, dSETDB1 expression did not change in these mutants as measured with a Hemagglutinin (HA) tag knocked into the endogenous *dSETDB1* locus (*dSETDB1-HA*) (S2K–S2L1 Fig) [30]. Thus, we conclude that *dWnt4* acts downstream of *dSETDB1* to promote CB differentiation.

### *dWnt4* is expressed in the escort cells and acts in an autocrine manner to promote CB differentiation

**Fig 2:**
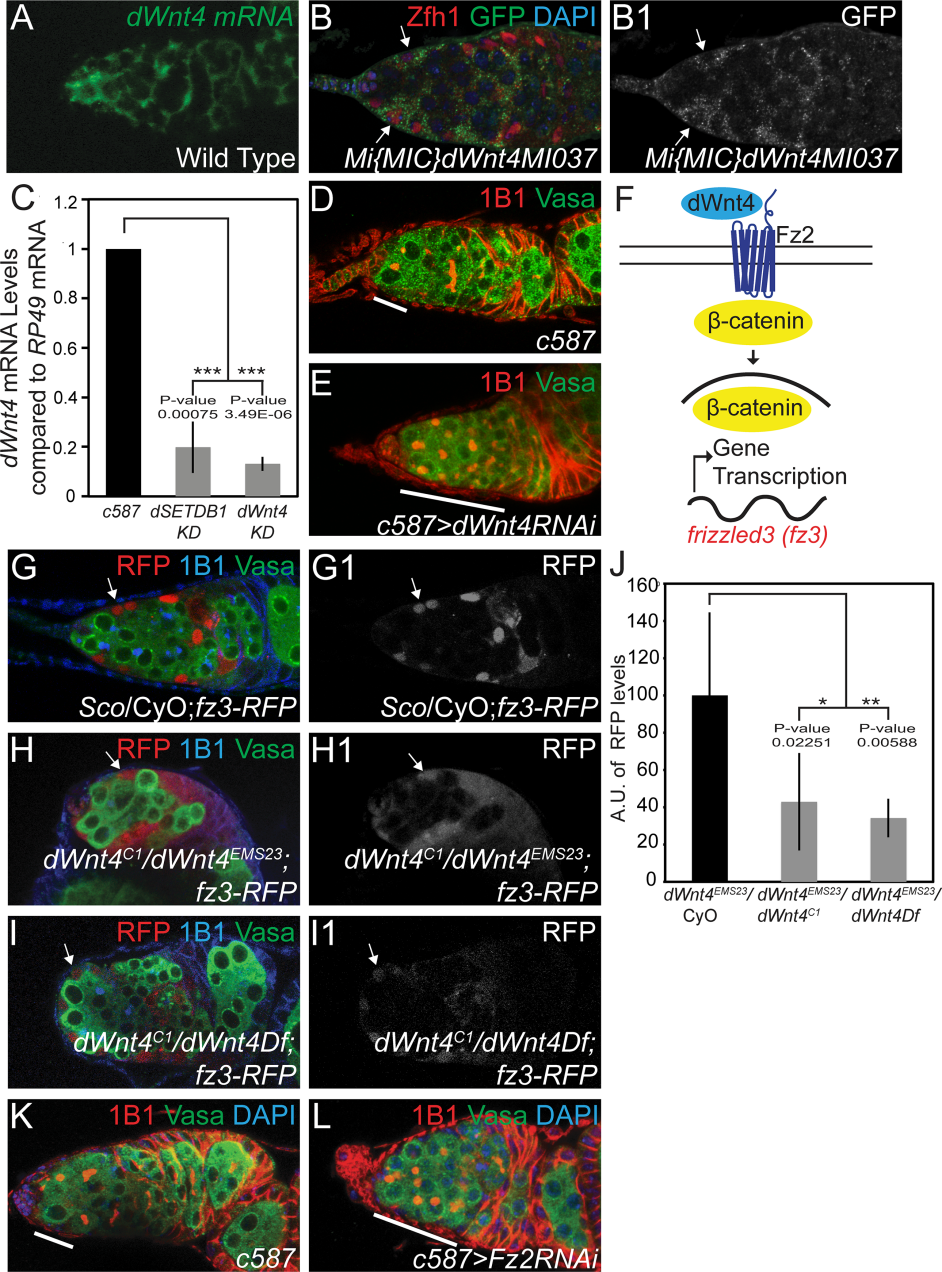
*dWnt4* is expressed in and acts from the escort cells. (A) Fluorescent *in situ* hybridization (FISH) for *dWnt4* mRNA (green) showing its expression in the somatic niche cells. (B-B1) Germarium of a *dWnt4* reporter stained for Zfh1 (red), GFP (green) and DAPI (blue) showing the expression of GFP in the escort cells (white arrows). (C) A qRT-PCR analysis showing *dWnt4* mRNA levels are downregulated in escort cell knockdowns (KD) of *dSETDB1* and *dWnt4*. (D-E) Control and *dWnt4 KD* stained with 1B1 (red) and Vasa (green) showing an accumulation of >3 undifferentiated cells in *dWnt4 KD* (white line). (F) A schematic of the dWnt4 pathway. dWnt4 binds to its receptor, Frizzled2 (Fz2) and activates a signaling cascade. This leads to stabilization of a transcription factor, β-catenin (Armadillo) and leads to activation of downstream target genes, such as *fz3*. (G-G1) Germarium of a fly carrying *frizzled3 RFP* (*fz3-RFP*) stained with RFP (red), 1B1 (blue) and Vasa (green) showing expression of Fz3RFP in the escort cells (white arrow). (H-I1) *dWnt4* mutants carrying the same transgene stained with RFP (red), 1B1 (blue) and Vasa (green) showing downregulation of Fz3-RFP expression (white arrow). (J) Quantification (n = 6) of RFP in the escort cells showing a downregulation in *dWnt4* mutants. (K-L) Control and *Frizzled2 KD*, stained with 1B1 (red), Vasa (green) and DAPI (blue) showing an accumulation of >3 undifferentiated cells in *Frizzled2 KD* (white line).

If *dSETDB1* is regulating CB differentiation from the somatic cells via *dWnt4* then: 1) *dWnt4* should be expressed in the somatic cells, 2) Loss of *dSETDB1* in the somatic cells should eliminate *dWnt4* expression, and 3) Loss of *dWnt4* in the somatic cells should prevent CB differentiation. Using fluorescent *in situ* hybridization (FISH) in wild type germaria, we found that *dWnt4* is primarily expressed in the somatic cells that surround the germ line (Fig 2A). To independently verify this result, we used a GFP-containing Minos element inserted into the *dWnt4* promoter as a reporter. We observed GFP expression in the escort cells of the somatic niche (Fig 2B and 2B1). To determine if *dSETDB1* is required in the escort cells of the germarium to permit *dWnt4* expression, we utilized RNA interference (RNAi) driven by *c587-GAL4*, which is expressed in the escort cells, to knock down *dSETDB1* [31]. *dSETDB1* knockdown caused a significant reduction in *dWnt4* mRNA levels as measured using qRT-PCR (Fig 2C). Next, we determined if *dWnt4* is required in the escort cells for differentiation by depleting *dWnt4* using *c587-GAL4* to drive RNAi (Fig 2C). Loss of *dWnt4* specifically in the escort cells of the germaria resulted in the loss of CB differentiation (60%, n = 86) compared to the control (4%, n = 77) (Fig 2D and 2E) (Table 1). Depletion of *dWnt4* in the germ line using *nos*-*GAL4* or in the terminal filament and cap cells using *hedgehog (hh)-GAL4*, did not cause any loss of differentiation (S3A–S3E Fig) [32–34]. Thus, *dSETDB1* regulates *dWnt4* in the escort cells of germaria to promote CB differentiation.

The dWnt4 ligand is known to bind to the Fz2 receptor to induce a signaling cascade, but in which cell type does the dWnt4 from the escort cell cause a response? (Fig 2F) [19,22,35]. We considered two possibilities: 1) dWnt4 from the escort cells directly promotes CB differentiation by activating the receptors on the germline cell surface, or 2) dWnt4 from the escort cells indirectly causes CB differentiation by activating the receptors on the escort cell surface. To distinguish between these two possibilities, we monitored the expression of a downstream transcriptional reporter activated in response to Wnt signaling, namely the Frizzled3 (Fz3) promoter fused to RFP (Fz3RFP) [36]. Fz3RFP was primarily expressed in the escort cells but not in the germ line, and this expression was significantly reduced in *dWnt4* mutants (Fig 2G–2J). To further test if dWnt4 signals act on the escort cells, we selectively depleted the *Fz2* receptor by RNAi. We found that somatic escort cell depletion of *Fz2* results in an accumulation of undifferentiated cells (Fig 2K and 2L) (Table 1). Thus, *dWnt4* acts in an autocrine loop, being released from and acting on the escort cells to indirectly promote CB differentiation.

### dWnt4 regulates escort cell encapsulation of CB to promote differentiation

**Fig 3:**
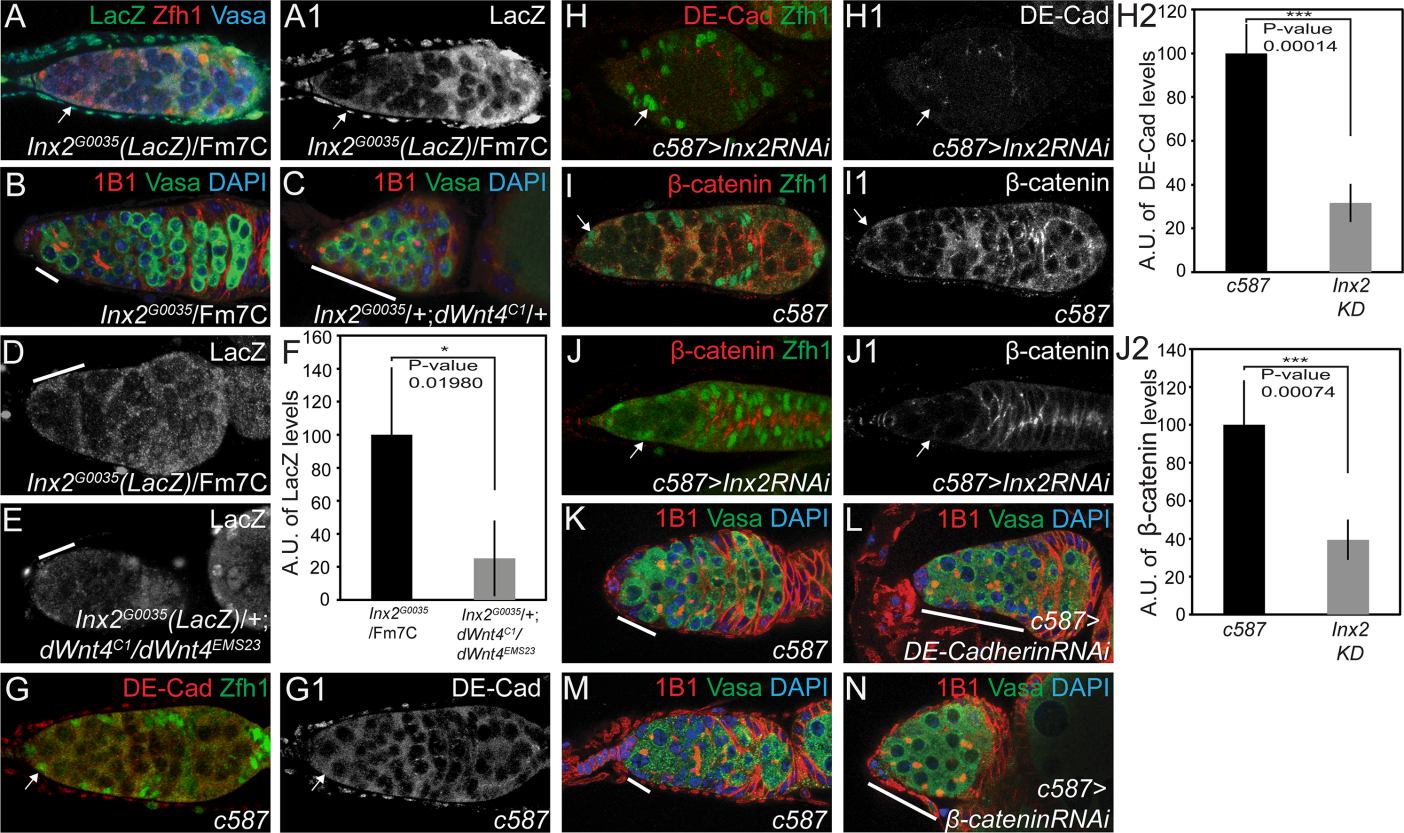
dWnt4 regulates Innexin2 in the escort cells. (A-A1) Germarium of Innexin2-LacZ stained for LacZ (green), Zfh1 (red) and Vasa (blue) showing Inx2 expression in escort cells (white arrow). (B-C) *inx2* heterozygote and *inx2;dWnt4* trans-heterozygote stained with 1B1 (red), Vasa (green) and DAPI (blue) showing an accumulation of >3 undifferentiated cells in the trans-heterozygote (white line). (D-E) *inx2* heterozygote and *inx2;dWnt4* mutant germaria stained with LacZ (white line) showing perturbed LacZ expression in *dWnt4* mutant (white line). (F) Quantification (n = 5) of LacZ in the escort cells showing a downregulation in *dWnt4* mutant. (G-J2) *c587-GAL4* and escort cell knockdown (KD) of *inx2* stained for DE-Cadherin (red) and β-catenin (red) respectively, and Zfh1 (green) (white arrow) showing perturbed DE-Cadherin and β-catenin expression in *inx2 KD*. Quantifications for the same shown in H2 and J2. (K–M) Control, *DE-Cadherin KD* and *β-catenin KD* stained with 1B1 (red), Vasa (green) and DAPI (blue) respectively, showing an accumulation of >3 undifferentiated cells in *DE-Cadherin KD* and *β-catenin KDs* (white line).

The observation that dWnt4 acts in the escort cells was intriguing, as dWnt4 signaling is known to regulate cell adhesion [37,38], a critical function for encapsulating CBs and promoting their differentiation [9,39]. The gap junction protein Inx2 is required in the escort cells for differentiation and *inx2* has been shown to regulate accumulation of DE-Cadherin (DE-Cad) [40]. As *wg*, a member of the Wnt signaling pathway, regulates *inx2* in the developing foregut [41], we hypothesized that *dWnt4* could regulate *inx2* in the escort cells to mediate DE-Cad based adhesion for encapsulating CBs. To test this, we first monitored the wild type expression of Inx2 using an *inx2 LacZ* reporter and found it to be expressed in the somatic niche, including escort cells (Fig 3A and 3A1) [41]. Next, we analyzed whether *dWnt4* and *inx2* act through the same pathway to promote CB differentiation by genetically removing a copy of *dWnt4* and *inx2* to create double heterozygous flies, and staining the ovaries for Vasa and 1B1. Undifferentiated cells accumulated in the trans-heterozygous germaria, as compared to heterozygous controls (10% in *dWnt4* heterozygotes, n = 40; 14% in *inx2* heterozygotes n = 57; 78% in transheterozygous n = 57) (Fig 3B and 3C) (Table 1). We found that LacZ levels, a reporter of *inx2* transcription, were reduced in escort cells of *dWnt4* mutant germaria compared to the control (Fig 3D and 3F). These results support the notion that *inx2* acts in the same pathway as *dWnt4* in the escort cells to promote CB differentiation.

*inx2* mutants accumulate undifferentiated spectrosome-containing cells [40]. These accumulated, undifferentiated cells show reduced DE-Cad accumulation where they abut the follicle cells [40]. To determine if loss of *inx2* in the escort cells also leads to perturbed DE-Cad levels, we depleted *inx2* in the escort cells using *c587-GAL4* and found that DE-Cad levels were altered compared to control (Fig 3G–3H2). Because DE-Cad is part of the AJ complex, we also monitored for another critical AJ component protein, β-catenin. We found that compared to control, β-catenin levels, like the DE-Cad levels, were perturbed in escort cells depleted for *inx2* (Fig 3I–3J2). Selective RNAi depletion of *DE-Cad* and *β-catenin* in the escort cells using *c587-GAL4* resulted in an accumulation of CBs as compared to the control (Fig 3K–3N and Table 1). This suggests that *inx2* regulates AJ component proteins DE-Cad and β-catenin to promote CB differentiation.

**Fig 4:**
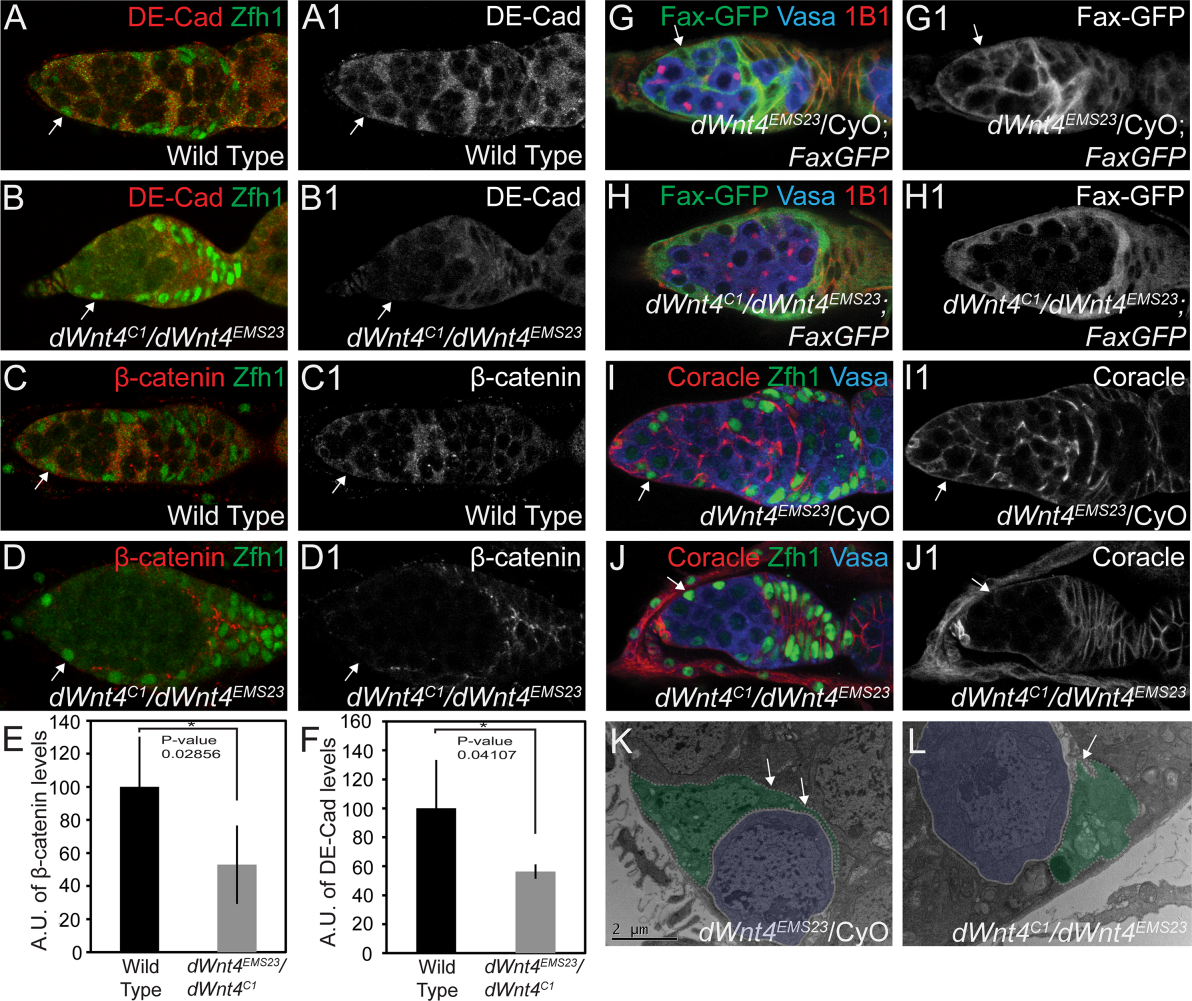
dWnt4 regulates encapsulation of the CB via adherens junction proteins. (A-B1) Wild type and *dWnt4* mutant germaria stained with DE-Cadherin (red) and Zfh1 (green) (white arrow) show perturbed DE-Cadherin expression in *dWnt4* mutants. (C-D1) Wild type and *dWnt4* mutants stained with β-catenin (red) and Zfh1 (green) (white arrow) display perturbed β-catenin expression in *dWnt4* mutants. (E-F) Quantification (n = 5) of DE-Cadherin and β-catenin in the escort cells reveals a downregulation in *dWnt4* mutants. (G-H1) *dWnt4* heterozygote and *dWnt4* mutant germaria stained with GFP (green), Vasa (blue) and 1B1 (red) showing a loss of encapsulation in the *dWnt4* mutant. (I-J1) *dWnt4* heterozygote and *dWnt4* mutant germaria stained with Coracle (red), Vasa (blue) and Zfh1 (green) (white arrow) showing a loss of encapsulation in the *dWnt4* mutant. (K-L) Transmission Electron Micrography of *dWnt4* heterozygote showing encapsulation (white arrows) and *dWnt4* mutant showing loss of encapsulation. CB is highlighted in blue and escort cell is highlighted in green.

Escort cell encapsulation of CBs is required for CB differentiation, but the escort cell intrinsic pathways that regulate this process have not been fully elucidated [9,39]. We hypothesized that Wnt signaling may promote CB differentiation by modulating AJ proteins via *inx2* in the escort cell to promote encapsulation of the CB. To determine if *dWnt4* regulates adhesion, we monitored the expression of the AJ components DE-Cad and β-catenin and found their expression was perturbed in *dWnt4* mutants, as compared to controls (Fig 4A–4F). To determine if *dWnt4* regulates escort cell encapsulation of CB, we visualized the cytoplasmic processes of the escort cell by labeling them with Fax (Failed axon-connections) tagged to GFP (FaxGFP) and antibodies against Coracle (Cora) [8,42]. Encapsulation was lost in *dWnt4* mutants as compared to control germaria (Fig 4G–4J1). As a further test, we carried out transmission electron microscopy (TEM) and found that *dWnt4* mutants failed to encapsulate CBs compared to WT germaria (Fig 4K and 4L). This was not due to a loss of escort cell fate specification or cell death, as monitored respectively by escort cell marker Zfh1 and cleaved Caspase 3 staining (S4A–S4D Fig) [42]. Somatic depletion of *dWnt4* did result in a 13% reduction in the number of Zfh1 positive cells (13 ± 2 in *dWnt4* mutants, n = 30, compared to 15 ± 2 in *c587-GAL4*, n = 30, P-value = 0.004). However, these escort cells all lost their ability to encapsulate the CBs (Fig 4G–4H1). Lastly, we found that depleting *inx2* and AJ components, *DE-Cad* and *β-catenin*, in the escort cells led to a similar loss of encapsulation as seen in the *dWnt4* mutants (S4E–S4H1 Fig). Thus, *dWnt4* promotes encapsulation of CBs through regulation of *inx2* and AJ components.

### Upregulation of transposons leads to downregulation of dWnt4 signaling

As loss of *dSETDB1* results in loss of *dWnt4*, we predicted that loss of *dSETDB1* would also result in changes to AJ protein mediated encapsulation. We monitored DE-Cad and β-catenin in *dSETDB1* mutants: both were downregulated in the *dSETDB1* germaria compared to the control (S5A–S5F Fig). *dSETDB1* mutants also showed downregulation of the Wnt reporter Fz3RFP and displayed a loss of escort cell encapsulation, as assessed with antibodies against FaxGFP and Cora (S5G–S5M2 Fig) [8,42]. Although we observed a reduction in the number of Zfh1 positive cells in *dSETDB1* (7 ± 3 in *dSETDB1* mutants, n = 20 compared to 15 ± 2 in *c587-GAL4*, n = 30, P-value = 9.2x10E-14), all the escort cells present at the niche were clearly incapable of encapsulation (S5J–S5M2 Fig). Therefore, we conclude that *dSETDB1* acts via *dWnt4* to permit CB differentiation by regulating the levels of AJ proteins in escort cells needed to promote CB encapsulation.

**Fig 5:**
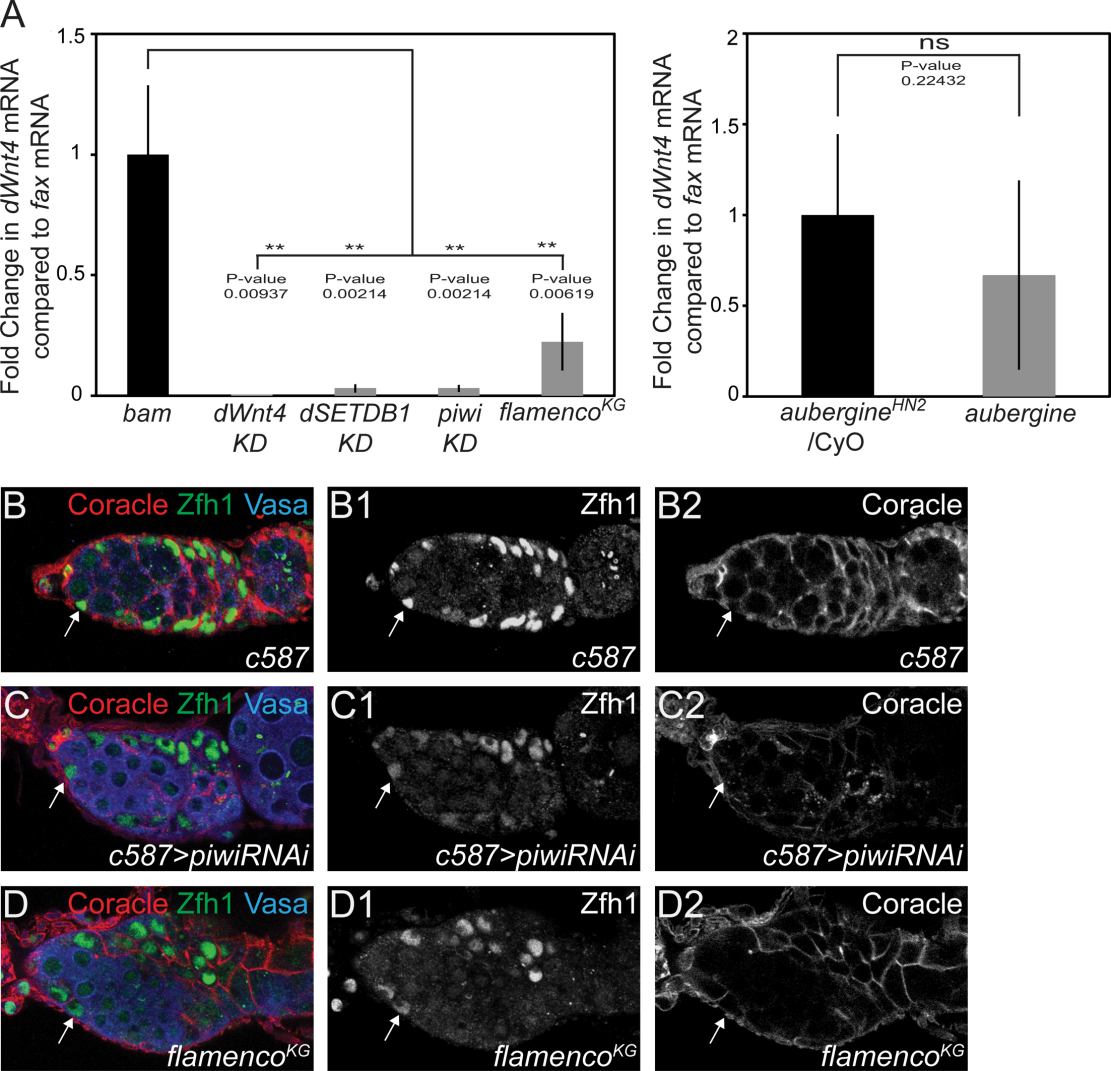
Upregulation of transposons leads to downregulation of *dWnt4*. (A) qRT-PCR analysis showing a significant downregulation of *dWnt4* mRNA levels compared to escort cell specific *fax* mRNA levels in escort cell specific knockdowns (KD) of *dWnt4*, *dSETDB1*, *piwi and flamenco* mutant compared to *bam* mutants. No significant change in *dWnt4* mRNA levels compared to *fax* mRNA levels was seen between *aubergine* mutants and its heterozygous control. (B–D2) Control, *piwi KD* and *flamenco* stained with Coracle (red), Zfh1 (green) (white arrow) and Vasa (blue) showing loss of CB encapsulation in *piwi KD* and *flamenco* compared to control.

Finally, we asked if *dSETDB1* regulates *dWnt4* through TEs via its role in the piRNA pathway [11]. We predicted that, if this was the case, mutations in the piRNA pathway that upregulate TEs should analogously display downregulation of *dWnt4*. This hypothesis is supported by the fact that mutations affecting the piRNA pathway in the somatic niche show similar differentiation defects [11,16,17]. We monitored the level of *dWnt4* mRNA in escort knockdown of *piwi (piwi KD)*, *flamenco* and *aubergine* mutants. The combination of these mutants allows us to test in which cell type the production of TEs results in *dWnt4* downregulation, as they all affect the soma and germ line differently. Mutations in *piwi KD* and the *flamenco* piRNA cluster upregulate TEs only in the somatic cells while *aubergine* (*aub)* upregulate TEs only in the germ line [43–45]. *flamenco* mutants also have the advantage of altering piRNA production just through a mutation in the genomic region that encodes piRNAs and not through a block of a processing protein that could have more pleiotropic effects [44–46]. We compared *dWnt4* mRNA levels in these three mutants to a housekeeping gene, *RP49*, and an escort cell marker, *fax*. We found using qRT-PCR that only mutations upregulating TEs in the soma but not in the germ line downregulated *dWnt4* (Fig 5A) (S6A Fig). Additionally, we carried out *in situ* hybridization for *dWnt4* and monitored the *dWnt4* reporter and found that *dWnt4* was downregulated in escort cells of *dSETDB1 KD*, *piwi KD* and *flamenco* mutants compared to wild type (S6B–S6I2 Fig). We also found that, like *dSETDB1* mutants, *piwi KD* and *flamenco* downregulated β-catenin and DE-Cad (S7A–S7J Fig). Additionally, *piwi KD* showed downregulation of Fz3RFP in the escort cells (S7K and S7M Fig). Next, we monitored escort cell encapsulation using antibodies against Cora [42]. Similar to *dSETDB1* and *dWnt4* mutants, *piwi KD* and *flamenco* mutants showed an overall reduction in number of escort cells (6 ± 3 in *piwi KD*, n = 31, P-value = 2.6x10E-18 and 10 ± 3 in *flamenco* mutants, n = 40, P-value = 1.8x10E-09 compared to 15 ± 2 in *c587-GAL4*, n = 30), but the escort cells present were defective in encapsulation of the CB (Fig 5B–5D2). Although it was previously reported that *dWnt4* mutants exhibited a reduction in *piwi* mRNA and protein levels, we found that *piwi* mRNA and protein levels were not altered in *dWnt4* mutants (S8A–S8D Fig). This data suggests that that *dWnt4* acts downstream of TEs and piRNA pathway to promote CB differentiation in the escort cells.

**Fig 6:**
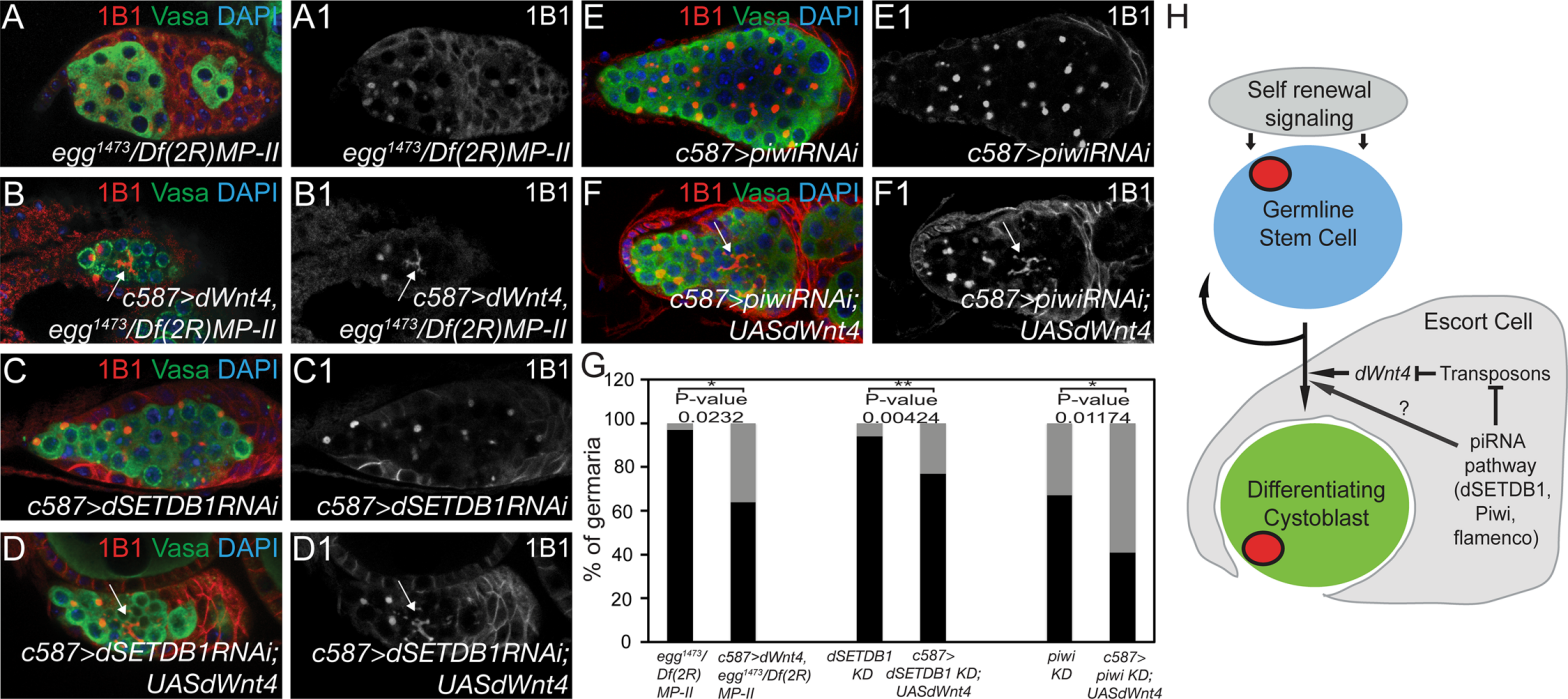
Overexpression of *dWnt4* in the escort cell partially rescues differentiation defects in piRNA pathway mutants. (A-A1) *dSETDB1* mutant stained for 1B1 (red), Vasa (green) and DAPI (blue) showing an accumulation of undifferentiated cells and a lack of fusomes (3% exhibited 16-cell cysts, n = 29). (B-B1) *dSETDB1* mutant stained for 1B1 (red), Vasa (green) and DAPI (blue) where *dWnt4* has been overexpressed in the escort cells showing presence of fusomes (white arrow) (36% exhibited 16-cell cysts, n = 14). (C-C1) Germarium where *dSETDB1* has been specifically knocked down (KD) in the escort cells stained for 1B1 (red), Vasa (green) and DAPI (blue) showing an accumulation of undifferentiated cells and a lack of fusomes (6% exhibited 16 cell-cyst, n = 50). (D-D1) Germarium of *dSETDB1 KD* and *dWnt4* overexpression in the escort cells stained for 1B1 (red), Vasa (green) and DAPI (blue) showing presence of fusomes (white arrow) (23% exhibited 16-cell cysts). (E-E1) Germarium of *piwi KD* stained for 1B1 (red), Vasa (green) and DAPI (blue) showing an accumulation of undifferentiated cells and a lack of fusomes (9% exhibited 16 cell-cyst, n = 40). (F-F1) Germarium of *piwi KD* and *dWnt4* overexpression in the escort cells showing a presence of fusomes (white arrow) (32% exhibited 16-cell cysts, n = 54). (G) Quantification of the *dSETDB1*, *dSETDB1 KD* and *piwi KD* showing a partial rescue of differentiation upon overexpression of *dWnt4* specifically in the escort cells. Black bars illustrate the percentage of germaria containing only spectrosomes; Gray bars illustrate the percentage of germaria that also contain fusomes. (H) A schematic showing *dWnt4* is expressed in the escort cells and regulates formation of cytoplasmic processes that encapsulates the CB promoting differentiation. *dWnt4* acts downstream of the piRNA pathway and is sensitive to transposons expression.

To test this more directly, we overexpressed *dWnt4* in piRNA mutants to see if it would rescue the CB differentiation phenotype. We used *c587-GAL4* to drive UASt *dWnt4* in *dSETDB1* mutants and stained them with Vasa and 1B1 [47]. Additionally, we depleted *dSETDB1* and *piwi* only in the somatic cells, while simultaneously expressing *dWnt4* there. We found that overexpression of *dWnt4* in the soma of these mutant germaria resulted in the formation of fusomes, a hallmark of differentiation (Fig 6A–6G). However, the rescue was only partial, as the *dWnt4* expressing *dSETDB1* mutants were still sterile and exhibited loss of later differentiated stages. Thus, we conclude that *dSETDB1* regulates *dWnt4* via TEs to modulate CB differentiation.

## Discussion

Our results indicate that *dSETDB1* controls the expression of *dWnt4* to facilitate differentiation. We find that *dWnt4* is expressed in the escort cells and acts on these same cells to promote CB differentiation. We have identified *inx2* and proteins of the AJ complex, DE-Cad and β-catenin, as downstream targets of *dWnt4*. These proteins regulate the encapsulation of the CB by the escort cells via altering the adhesion of the soma to the germ line to control differentiation (Fig 6H). We find that mutations in the piRNA pathway downstream of *dSETDB1* also downregulate *dWnt4* expression, suggesting that TEs modulate the expression of this signaling pathway through an as yet unidentified mechanism (Fig 6H). Additionally, the overexpression of *dWnt4* in the soma of these mutant germaria only partially rescued differentiation, suggesting that there are yet unknown factors that function in parallel to dWnt4 downstream of the piRNA pathway to regulate CB differentiation (Fig 6H).

Our findings are consistent with recently published work that shows *dWnt4* acts in an autocrine manner from the escort cells to promote germ cell differentiation. Wang *et al* find that Wnt4 along with Wnt2 signaling acts through the canonical pathway to promote proliferation and formation of escort cell processes [48]. They identify reactive oxygen species (ROS) as a main target of Wnt signaling, which maintains reduced redox state to promote CB differentiation. We find that TEs modulate *dWnt4* levels in the escort cells to regulate inx2 and AJ proteins, which are then required for CB encapsulation. Thus, we both independently identified separate pathways downstream of *dWnt4* in the escort cells, which promote GSC differentiation. Intriguingly, it has been shown that low redox levels are required to maintain DE-Cad levels [49,50]. We speculate that downstream of *dWnt4*, both ROS and *inx2* could be acting in parallel to regulate AJ proteins to promote differentiation via escort cell encapsulation.

Wang *et al* find a reduction in number of escort cells in germaria with abrogated Wnt signaling [48]. Analogously, we find a 13% reduction in *dWnt4* germaria. However, we find that these escort cells present at the niche do not extend processes that promote differentiation. Wang *et al*’s data shows that overexpression of Wnt signaling leads to an increase in number of escort cells but this increase does not lead to germ cell differentiation defects. This suggests that the mere presence of escort cells is not sufficient to promote differentiation. Thus, in addition to its required role in maintaining escort cell proliferation, our data suggests that dWnt4 signaling also serves a critical function in driving encapsulation of the stem cell daughter.

Our findings and those of Wang *et al* [48], contradict published work showing that *piwi* lies downstream of *dWnt4* [51]. It was reported that *dWnt4* mutants lose Piwi expression in escort cells and upregulate TEs. However, we find no change in Piwi levels, TE levels or double strand breaks in *dWnt4* mutants. We suggest two reasons for this discrepancy. First, Hamada-Kawaguchi *et al*, used semi-quantitative PCR to measure TE levels, as compared to our use of qRT-PCR [51]. Second, the altered morphology of escort cells in *dWnt4* mutants could make them difficult to find, perhaps explaining Hamada-Kawaguchi *et al*’s perceived loss of Piwi staining in the escort cells of *dWnt4* mutants. Thus, our work has discovered dWnt4 signaling acts downstream of the piRNA pathway to control CB differentiation.

Cohen *et al* [22], have reported that *dWnt4* is essential for the ensheathment of the entire ovariole in which germline stem cells reside. We find that upregulation of TEs results in *dWnt4* downregulation. However, *dSETDB1*, *piwi* and *flamenco* mutants have not been reported to exhibit disrupted ovariole ensheathment as seen in *dWnt4* mutants. This suggests that *dWnt4* acts to promote ovariole ensheathment independently of TEs at earlier developmental times.

TEs occupy a large part of our genome and controlling them is a critical task to protect genomic integrity [52]. It has been shown that the gypsy class of TEs, which are upregulated in somatic cells upon loss of the piRNA pathway, can infect the germ line to be passed on to the next generation [53–55]. We propose that TE-mediated modulation of *dWnt4* signaling in the somatic cells of the gonad acts to prevent the TE damaged eggs from being propagated by controlling somatic encapsulation of the germ line. Somatic encapsulation can act as a differentiation cue by either protecting the germ line from receiving self-renewal cues or by passing a differentiation cue through the gap junctions present between the soma and germ line [40,56,57]. Wnt signaling is a conserved signaling pathway whose dysregulation has been implicated in several human cancers [58]. Furthermore, some cancers have been associated with increased levels of TEs [52]. Our work suggests a chromatin-transposon-Wnt signaling axis could act as a driver of both differentiation and cancers by perturbing Wnt expression.

## Material and Methods

### Microarrays

Microarrays were performed at the NYU genomics core as described in [59] and analyzed at the CFG Core Facility, University at Albany SUNY using Genespring GX v12.6.

### Transmission electron microscopy

Transmission electron microscopy was performed at the NYU EM core as described in [60].

### Fly stocks

The following fly stocks were used in the study: *dWnt4*^*EMS23*^ *bw1/*CyO, *dWnt4*^*C1*^*/*CyO, *w1118; def (2L) Bsc291/*CyO, *egg*^*1473*^*/*CyO, *Df(2R)Dll-MP/*SM6a, *hs-bam*, *bamGFP*, *c587-GAL4*, *nos-GAL4*::*VP16*, y1 w*; Mi{MIC}Wnt4MI03717/SM6a, *Armadillo*RNAi (Bloomington 31304), *Shg*RNAi (Bloomington 32904), *Fz2*RNAi (27568), *Piwi*RNAi (Bloomington 33724), *flamenco*^*KG*^*/Fm7*, *faxGFP*, *W(67c23) P{w+mC] = lacW}Inx2[G0035]/Fm7c (*Bloomington), *aubergine*^*HN2*^*/*CyO (Bloomington); *aubergine*^*N11*^*/*CyO, *Sco/*CyO;MKRS/TM6 (Lehmann Lab); *dSETDB1HA* (Bontron Lab); *Sco/*Cyo;*Frz3RFP* (Bach Lab); *w-*,*P[w+UASdwnt4];P[w+UASdwnt4];P[w+UASdwnt4]* (Newfeld Lab; Pandur Lab); *Wnt4*RNAi (V104671), *Innexin2*RNAi (V102194) (VDRC); *egg*RNAi (TriP) and *dSETDB1HA*,*dWnt4*^*C1*^*/*CyO (This study).

### Collection and fixation of tissues

Collection and fixation of ovaries was carried out as previously described (de Cuevas et al., 1996).

### Immunostaining

Immunostaning of the ovaries was carried out with the following Primary antibodies: Rb Vasa (1:5000, this study), Ch Vasa (1:500, Lehmann Lab and this study), Ch GFP (1:1000, abcam AB13970), Rb pMAD (1:200, abcam AB52903), Mo BamC (1:200, DSHB), Rat HA (1:100, santa cruz sc53516, Mo β-catenin (Armadillo) (1:15, DSHB), Rat DE-CAD (DCAD2) (1:5, DSHB), Mo H4K20Me3 (1:500, abcam AB78517), Mo LacZ (1:50, Promega), Mo 1B1 (1:20, DSHB), Rb PH3 (1:200, Cell Signaling 97015), Rb Piwi (1:500, Lehmann Lab), Rb zfh1 (1:1000, Lehmann Lab), Rb Orb (1:500, Lehmann Lab), Mo Coracle (1:200, DSHB), Mo Lamin C (1:20, DSHB), Rb H2Av (1:500, Rockland 600-401-914), and Rb Caspase3 (1:300, Cell Signaling 96615).

Alexa 488 (Molecular Probes), Cy3 and Cy5 (Jackson Labs) conjugated secondary antibodies were used at a concentration of 1:500.

### Fluorescence *in situ* hybridization

FISH of the ovaries was carried out with a mixture of 48 probes against *dWnt4*, which were obtained from Stellaris FISH probes (Custom assay with quasar 570 dye, SMF-1063-5). The ovaries were dissected in PBS, fixed in 4% formaldehyde in PBS for 30 minutes and washed 3 times with PBT (0.3% TritonX 100). Next, they were treated with 3ug/ml Proteinase K in PBS and incubated on ice for an hour. The tissue was blocked in 2mg/ml glycine in PBT twice for 2 minutes each, rinsed twice with PBT. The ovaries were fixed again for 30 minutes in 4% formaldehyde in PBS for 30 minutes at room temperature. The tissue was then washed with PBT 5 times for 2 minutes and washed with fresh pre-hybridization mix (10% deionized formamide in 2x SSC) for 10 minutes at 37°C. 60ul per sample of hybridization mix (10% deionized formamide, 1mg/ml of t-RNA and sperm ssDNA, 50 ng/ul probe, 10% Dextran sulphate, 2mg/ml BSA, 2x SSC and 2ul of RNase In) was added and the sample was incubated overnight at 37°C. The sample was washed in pre-hybridization mix (warmed at 37°C) for 15 minutes, and thrice with 1x PBS for 45 minutes each and mounted.

### Fluorescence imaging

The tissues were visualized under 10X, 20X, 40X and 63x objective lenses. The images were acquired using a Zeiss LSM-510/710 confocal microscope under 40X and 63x objective.

### Quantification analysis

In order to calculate intensities for LacZ, DE-Cadherin, β-catenin, Piwi and Fz3RFP, both, control and mutant germaria were taken under the same confocal settings and Z stack planes were obtained. For DE-Cadherin and β-catenin quantification, specific planes showing both the CB and Zfh1 positive escort cell were chosen and the area of Zfh1 positive surrounding the CB was outlined. For Fz3-RFP and Piwi quantification, the Zfh1 positive cell was outlined. The intensity of this region was analyzed using ImageJ. The ratio between mean and area was calculated. Average of all these ratios, per image was calculated and a comparison was made between control and mutants. P-value was determined by two-tailed equal variance t test by comparing the intensities of mutants vs. wild type strains. A minimum of 5 germaria were chosen for the each quantification.

### Materials and reagents

Fly food was created using the procedures from the Ruth Lehmann lab at NYU (summer/winter mix), and used to fill narrow vials to approximately 12mL.

### Real-time PCR

Expression levels of *dWnt4* in *dWnt4*, *dSETDB1*, *bam*, *piwi*, *flamenco*, and *aubergine* mutants were analyzed using real-time, quantitative PCR.

### RNA isolation

RNA was isolated using the Trizol reagent protocol. The ovaries were first homogenized in Trizol and kept for 5 minutes at room temperature. Choloform was added and the sample was centrifuged at 12000g for 15 minutes at 4°C. The aqueous phase was aspirated out in a new tube. 100% isopropanol was added and the sample was incubated for 10 minutes at room temperature, followed by centrifugation at 12000g for 10 minutes at 4°C. The supernatant was removed and the RNA pellet was washed with 75% ethanol at 7500g for 5 minutes at 4°C. The wash was discarded and the pellet was allowed to air dry for 10 minutes and later re-suspended in RNase free water. The Turbo DNase (Life Technologies, Catalog Number: AM1907) protocol was used to remove any DNA contamination.

Reverse transcription was performed with Super Script III (Life Technologies, Catalog Number: 1808051) The cDNA samples were diluted to 50ng/μl. *dWnt4 and inx2* specific primer was used (IDT). All real-time PCR reactions were carried out using the ABI 7700 sequence detection system (Applied Biosystems) and the amplifications were done using the iTaq Universal Probes Supermix (Biorad, Catalog Number: 172–5130). The thermal cycling conditions consisted of 50°C for 2 min, 95°C for 10 min, 40 cycles at 95°C for 15s, and 60°C for 60s. The experiments were carried out in technical triplicate and three biological replicates for each data point.

To calculate fold change in *dWnt4 and piwi* mRNA levels compared to *fax* mRNA levels, ratio and then average of the same was calculated between the two for each of the three biological replicate. Error bars were plotted using Standard deviation of the ratios. P-value was determined by one-tailed equal variance t test by comparing ratios of mutants vs. wild type strains.

To calculate fold change in *dWnt4 and piwi* mRNA levels to *RP49* mRNA levels, average of the 2^ΔCt for three biological replicates was calculated. Error bars were plotted using Standard deviation of the ratios. P-value was determined by one-tailed equal variance t test by comparing ratios of mutants vs. wild type strains.

### Primers used for qRT-PCR

#### The following primers were used to perform qRT-PCR

F-Wnt4 (5’-CAACTGTGGCACCCTGTGC-3’), R-Wnt4 (5’-GTTCTCCAGCCGCTGGCAG-3’), F-Piwi (5’-CTTTGGACAAACTCCGAAATCAA-3’), R-Piwi (5’-GCAGCGGCTGATTGTGTCG-3’), F-ZAM (5’-TCCGACCTTCGATGGTACTC-3’), R-ZAM (5’-GTCGGTACCCCTAATGAGCA-3’), F-Idefix (5’-AAACAAATCGTGGCAGGAAG-3’), R-Idefix (5’-GCTTCTTTGGTTGGTCTGGA-3’), F-Fax (5’-CGTTCATCGAGCTGAATGGG-3’), R-Fax (5’-CCCGAGTCCAGGTACTTCT-3’), F-RP49 (5’-ATGACCATCCGCCCAGCATAC-3’) and R-RP49 (5’-CTGCATGAGCAGGACCTCCAG-3’).

## Supporting Information

S1 Fig *dWnt4* affects differentiation at the CB stage. (A) GO term analysis of *dSETDB1* mutants compared to *bam* mutants showing Wnt signaling to be primarily downregulated. (B-C) *dWnt4* mutants stained with 1B1 (red), Vasa (green) and DAPI (blue) showing pleotropic differentiation defect and loss of later stages. (D-E) *dWnt4* heterozygote and *dWnt4* mutant stained for 1B1 (red) and Orb (green) (white arrow) showing an oocyte specification defect. (F-G) Zoomed images (63X) of *dWnt4* heterozygote and *dWnt4* mutant stained for pMad (red) (white asterisk), BamC (green) and Vasa (blue) showing an accumulation of undifferentiated CBs. (H-I) *dWnt4* heterozygote and *dWnt4* mutant stained for LaminC (red) and Vasa (green) showing no increase in the somatic niche size (white arrow). (J-K) *dWnt4* heterozygote and *dWnt4* mutant stained for PH3 (red), Vasa (green) and DAPI (blue) showing similar cell division rate. (L-M) *dSETDB1* heterozygote and *dWnt4*, *dSETDB1* trans-heterozygote stained with 1B1 (red), Vasa (green) and DAPI (blue) showing an accumulation of >3 undifferentiated single cells (white line).
(TIF)

S2 Fig *dWnt4* acts downstream of *dSETDB1*. (A-B) *c587-GAL4* and escort cell knock down (KD) of *dSETDB1* stained with H4K20me3 (red) and Vasa (green) showing loss of heterochromatin expression in the soma (white arrow). (C-D) *dWnt4* heterozygote and *dWnt4* mutant stained with H4K20me3 (red) and Vasa (green) showing similar heterochromatin expression in the soma (white arrow). (E-F) *dWnt4 KD* do not show an upregulation of ZAM and Idefix levels compared to *dSETDB1 KD*. (G-H) *dSETDB1* heterozygote and *dSETDB1* mutant stained with H2Av (red), and Vasa (green) showing upregulated H2Av expression in the somatic cells (white line). (I-J) *dWnt4* heterozygote and *dWnt4* mutant stained with H2Av (red), 1B1 (blue) and Vasa (green) showing similar H2Av expression during meiosis (white line). (K–L1) *dWnt4* heterozygote and *dWnt4* mutant stained for dSETDB1 that is tagged with HA (red) and Vasa (green) showing similar dSETDB1 expression. The accumulation of dSETDB1 in *dWnt4* mutant is due to accumulation of undifferentiated CBs.
(TIF)

S3 Fig *dWnt4* is not required in the germ line or in the terminal filament and cap cells for differentiation. (A-B) *nos-GAL4* and *dWnt4* RNAi where *dWnt4* has been specifically knocked down in the germ line, stained with 1B1 (red), Vasa (green) and DAPI (blue) showing 2–3 undifferentiated cells. (C-D) *hh-GAL4* and *dWnt4* RNAi where *dWnt4* has been specifically knocked down in the terminal filament and cap cells, stained with 1B1 (red), Vasa (green) and DAPI (blue) showing 2–3 undifferentiated cells. (E) Quantification of the percentage of *nos-dWnt4* and *hh-dWnt4* RNAi germaria showing lack of differentiation defects.
(TIF)

S4 Fig Loss of AJ proteins leads to loss of CB encapsulation. (A-B) *dWnt4* heterozygote and *dWnt4* mutant stained for 1B1 (red) and Zfh1 (green) showing presence of escort cells (white arrows). (C-D) *dWnt4* heterozygote and *dWnt4* mutant stained for Caspase3 (red), Vasa (green) and 1B1 (blue) showing similar cell death. (E-H1) *c587-GAL4*, and escort cell knock down (KD) of *Inx2*, *DE-Cadherin* and *β-catenin* stained for Coracle (red), Vasa (green) and DAPI (blue) showing loss of encapsulation in *Inx2 KD*, *DE-Cadherin KD* and *β-catenin KD*.
(TIF)

S5 Fig *dSETDB1* mutants show loss of CB encapsulation. (A–B1) *c587-GAL4* and escort cell knock down (KD) of *dSETDB1* stained for DE-Cadherin (red), and Zfh1 (green) (white arrow) showing perturbed DE-Cadherin expression in *dSETDB1 KD*. (C) Quantification (n = 5) of DE-Cadherin levels in *c587-GAL4* and *dSETDB1 KD* showing a significant difference in *dSETDB1 KD*. (D-E1) *c587-GAL4* and *dSETDB1 KD* stained for β-catenin (red), and Zfh1 (green) (white arrow) showing perturbed β-catenin expression in *dSETDB1 KD*. (F) Quantification (n = 5) of β-catenin levels in *c587-GAL4* and *dSETDB1 KD* showing a significant difference in *dSETDB1 KD*. (G-I) Control and *dSETDB1 KD* stained for RFP (red), Zfh1 (green) (white arrow) and Vasa (blue) showing perturbed RFP expression in *dSETDB1 KD*. (J-K) Control and *dSETDBI* mutant stained with GFP (green), Vasa (blue), and 1B1 (red) showing loss of encapsulation in *dSETDB1* mutants. (L-M2) Control and *dSETDB1 KD* stained for Coracle (red), Zfh1 (green) (white arrow), and Vasa (blue) showing loss of encapsulation in *dSETDB1 KD*.
(TIF)

S6 Fig piRNA pathway mutants show downregulation of *dWnt4* in the escort cells. (A) qRT-PCR analysis showing a significant downregulation of *dWnt4* mRNA levels compared to *RP49* mRNA levels in escort cell specific knockdowns (KD) of *dWnt4 KD*, *dSETDB1 KD*, *piwi KD and flamenco* were compared to *bam* mutants. No significant change in *dWnt4* mRNA levels was observed compared to *RP49* mRNA levels was seen between *aubergine* mutants compared to its the heterozygous control. (B-F) Fluorescent *in situ* hybridization (FISH) for *dWnt4* mRNA in wild type, *dWnt4 KD*, *dSETDB1 KD*, *piwi KD* and *flamenco* showing downregulation of *dWnt4* in the soma compared to wild type. (G-G2) Germarium of a minos GFP (dWnt4 reporter) stained for Zfh1 (red), GFP (green) and 1B1 (blue) showing the expression of GFP primarily in the escort cells (white arrow). (H-I2) Germarium of *dSETDB1 KD* and *piwi KD* carrying dWnt4 reporter stained for Zfh1 (red), GFP (green) and 1B1 (blue) showing a downregulation of *dWnt4* in the escort cells.
(TIF)

S7 Fig piRNA pathway mutants show downregulation of β-catenin, DE-Cadherin and Fz3RFP levels. (A–C1) Wild type, escort cell knock down of *piwi (piwi KD)* and *flamenco* mutants respectively, stained for DE-Cadherin (red), and Zfh1 (green) (white arrow) showing perturbed DE-Cadherin expression in *piwi KD* and *flamenco* mutants. (D-E) Quantification (n = 5) of DE-Cadherin levels in wild type, *piwi KD* and *flamenco* mutants showing a significant decrease in mutants. (F–H1) Wild type, *piwi KD and flamenco* mutants stained for β-catenin (red), and Zfh1 (green) (white arrow) showing perturbed β-catenin expression in *piwi KD* and *flamenco* mutants. (I-J) Quantification (n = 5) of β-catenin levels in wild type, *piwi KD* and *flamenco* mutants showing a significant decrease in mutants. (K-K1) Germarium carrying a *fz3-RFP* transgene stained with RFP (red), Zfh1 (green) (white arrow) and Vasa (blue) showing expression of Fz3RFP in the escort cells. (L-L1) *piwi KD* carrying the same transgene stained with RFP (red), Zfh1 (green) (white arrow) and Vasa (blue) showing downregulation of Fz3 expression. (M) Quantification of RFP in the escort cells showing a significant downregulation in *piwi KD*.
(TIF)

S8 Fig *dWnt4* acts downstream of the piRNA pathway. (A) qRT-PCR analysis showing no significant change in *piwi* mRNA levels between *c587-GAL4* and germaria where *dWnt4* has been specifically depleted in the escort cells. (B–C1) *dWnt4* heterozygote and *dWnt4* mutant fly stained for Piwi (red) (white arrows), GFP (green) and 1B1 (blue) showing similar Piwi expression in the escort cells. (D) Quantification (n = 6) of Piwi in escort cells showing a no difference in *dWnt4* heterozygotes and *dWnt4* mutants.
(TIF)
