# Supplementary figures and images for: Transposon Dysregulation Modulates dWnt4 Signaling to Control Germline Stem Cell Differentiation in Drosophila
Source: PLoS Genet. 2016 Mar 28;12(3):e1005918. doi: 10.1371/journal.pgen.1005918 (PMC4809502; doi:10.1371/journal.pgen.1005918)

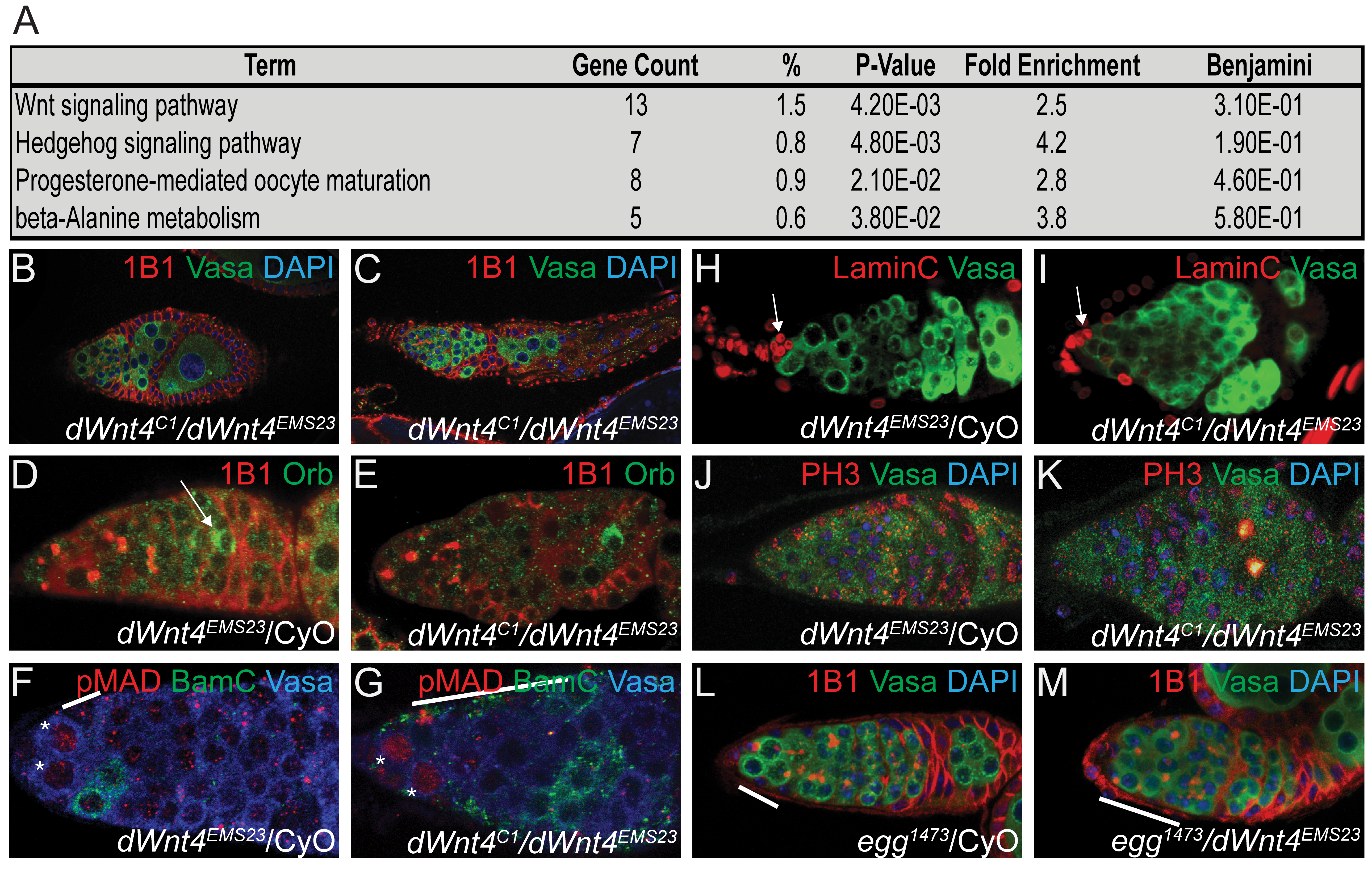

Supplement: S1 Fig — (A) GO term analysis of dSETDB1 mutants compared to bam mutants showing Wnt signaling to be primarily downregulated. (B-C) dWnt4 mutants stained with 1B1 (red), Vasa (green) and DAPI (blue) showing pleotropic differentiation defect and loss of later stages. (D-E) dWnt4 heterozygote and dWnt4 mutant stained for 1B1 (red) and Orb (green) (white arrow) showing an oocyte specification defect. (F-G) Zoomed images (63X) of dWnt4 heterozygote and dWnt4 mutant stained for pMad (red) (white asterisk), BamC (green) and Vasa (blue) showing an accumulation of undifferentiated CBs. (H-I) dWnt4 heterozygote and dWnt4 mutant stained for LaminC (red) and Vasa (green) showing no increase in the somatic niche size (white arrow). (J-K) dWnt4 heterozygote and dWnt4 mutant stained for PH3 (red), Vasa (green) and DAPI (blue) showing similar cell division rate. (L-M) dSETDB1 heterozygote and dWnt4, dSETDB1 trans-heterozygote stained with 1B1 (red), Vasa (green) and DAPI (blue) showing an accumulation of >3 undifferentiated single cells (white line). (TIF) [file pgen.1005918.s001.tif]

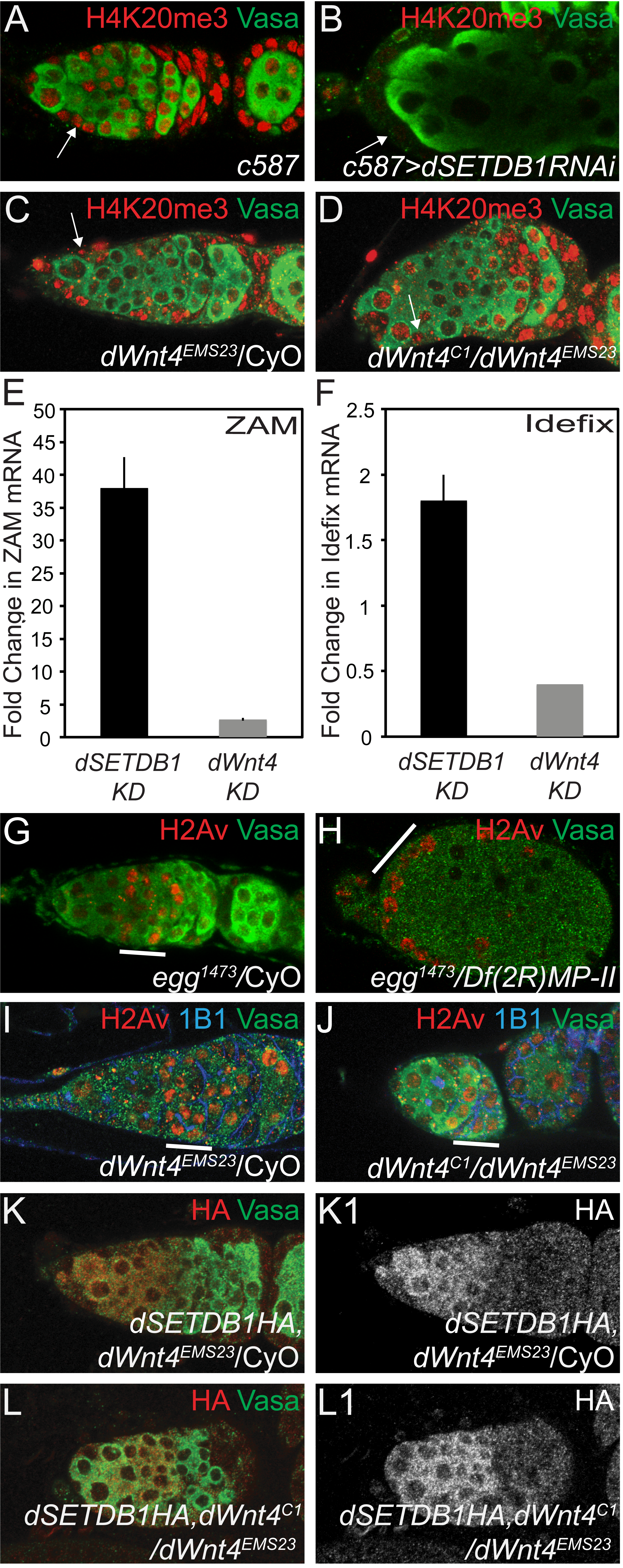

Supplement: S2 Fig — (A-B) c587-GAL4 and escort cell knock down (KD) of dSETDB1 stained with H4K20me3 (red) and Vasa (green) showing loss of heterochromatin expression in the soma (white arrow). (C-D) dWnt4 heterozygote and dWnt4 mutant stained with H4K20me3 (red) and Vasa (green) showing similar heterochromatin expression in the soma (white arrow). (E-F) dWnt4 KD do not show an upregulation of ZAM and Idefix levels compared to dSETDB1 KD. (G-H) dSETDB1 heterozygote and dSETDB1 mutant stained with H2Av (red), and Vasa (green) showing upregulated H2Av expression in the somatic cells (white line). (I-J) dWnt4 heterozygote and dWnt4 mutant stained with H2Av (red), 1B1 (blue) and Vasa (green) showing similar H2Av expression during meiosis (white line). (K–L1) dWnt4 heterozygote and dWnt4 mutant stained for dSETDB1 that is tagged with HA (red) and Vasa (green) showing similar dSETDB1 expression. The accumulation of dSETDB1 in dWnt4 mutant is due to accumulation of undifferentiated CBs. (TIF) [file pgen.1005918.s002.tif]

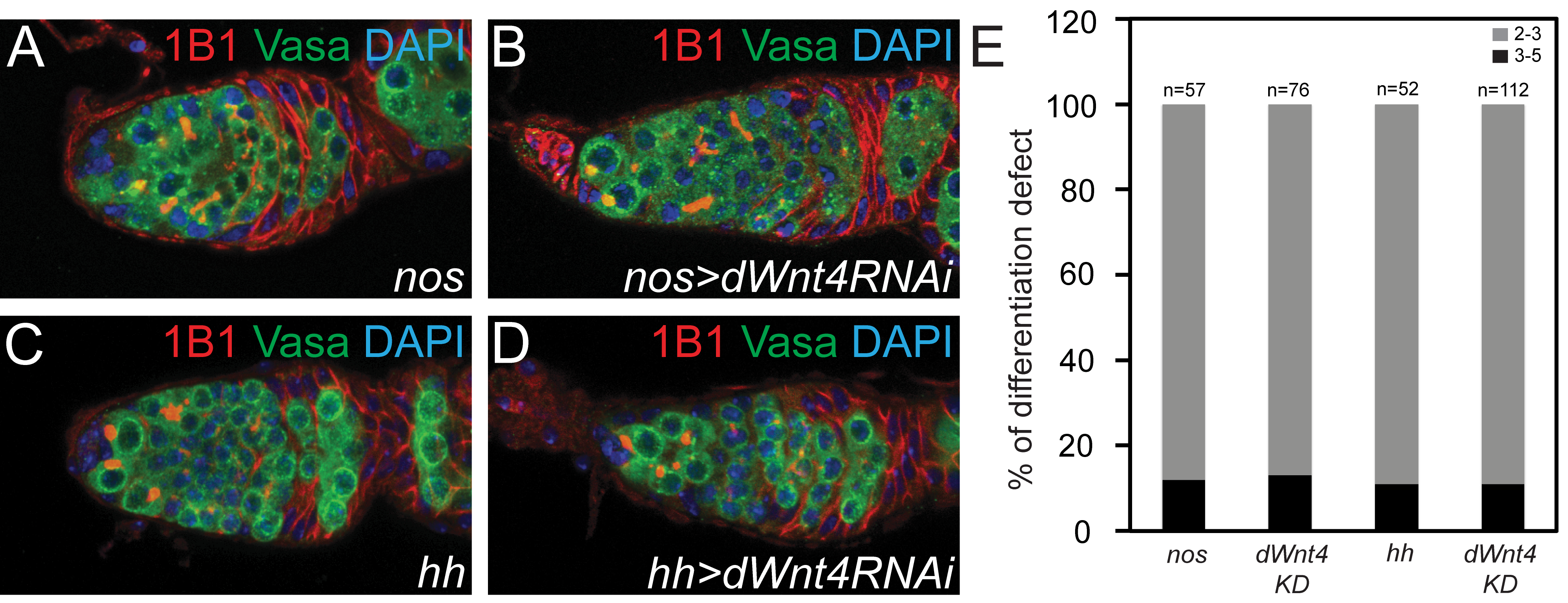

Supplement: S3 Fig — (A-B) nos-GAL4 and dWnt4 RNAi where dWnt4 has been specifically knocked down in the germ line, stained with 1B1 (red), Vasa (green) and DAPI (blue) showing 2–3 undifferentiated cells. (C-D) hh-GAL4 and dWnt4 RNAi where dWnt4 has been specifically knocked down in the terminal filament and cap cells, stained with 1B1 (red), Vasa (green) and DAPI (blue) showing 2–3 undifferentiated cells. (E) Quantification of the percentage of nos-dWnt4 and hh-dWnt4 RNAi germaria showing lack of differentiation defects. (TIF) [file pgen.1005918.s003.tif]

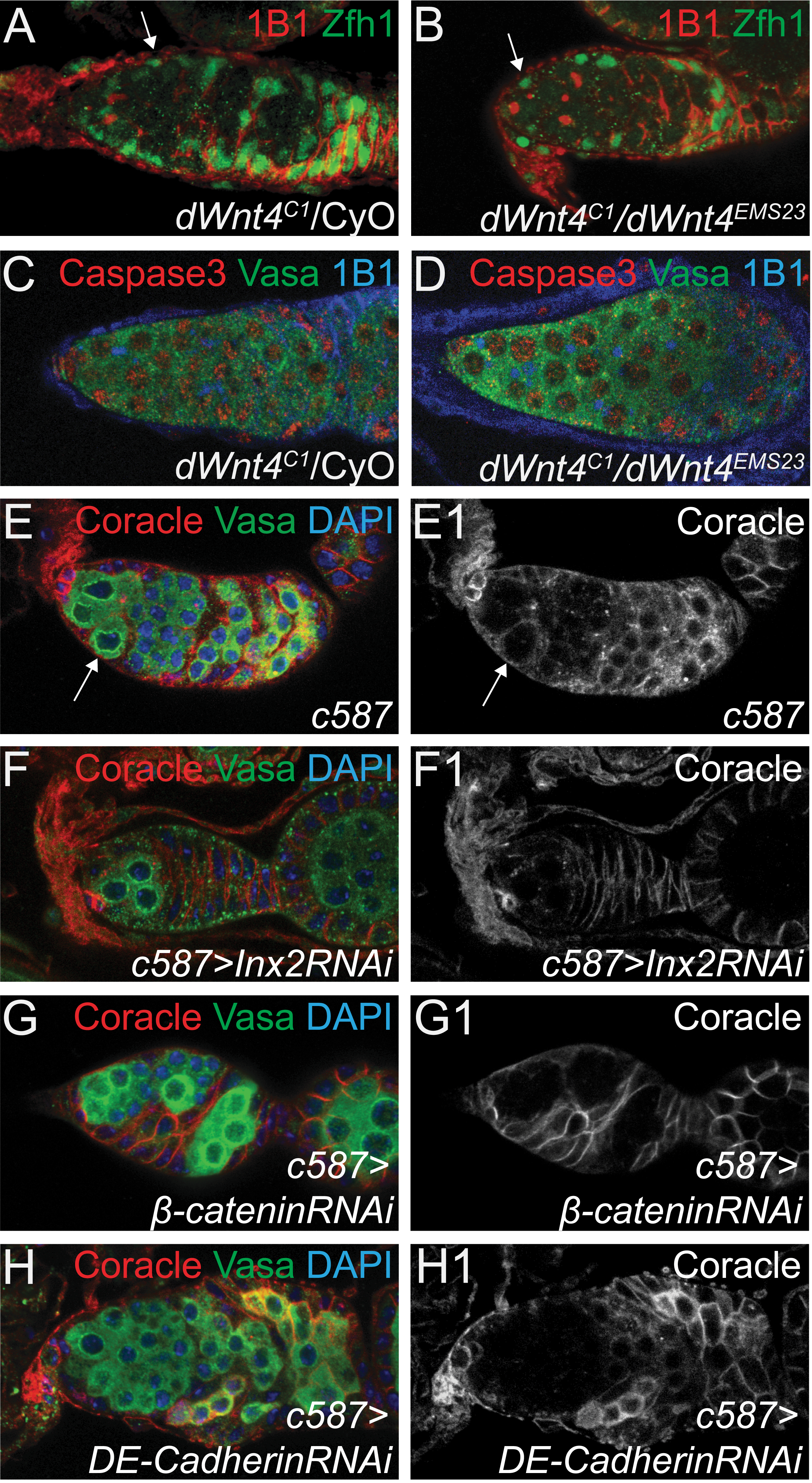

Supplement: S4 Fig — (A-B) dWnt4 heterozygote and dWnt4 mutant stained for 1B1 (red) and Zfh1 (green) showing presence of escort cells (white arrows). (C-D) dWnt4 heterozygote and dWnt4 mutant stained for Caspase3 (red), Vasa (green) and 1B1 (blue) showing similar cell death. (E-H1) c587-GAL4, and escort cell knock down (KD) of Inx2, DE-Cadherin and β-catenin stained for Coracle (red), Vasa (green) and DAPI (blue) showing loss of encapsulation in Inx2 KD, DE-Cadherin KD and β-catenin KD. (TIF) [file pgen.1005918.s004.tif]

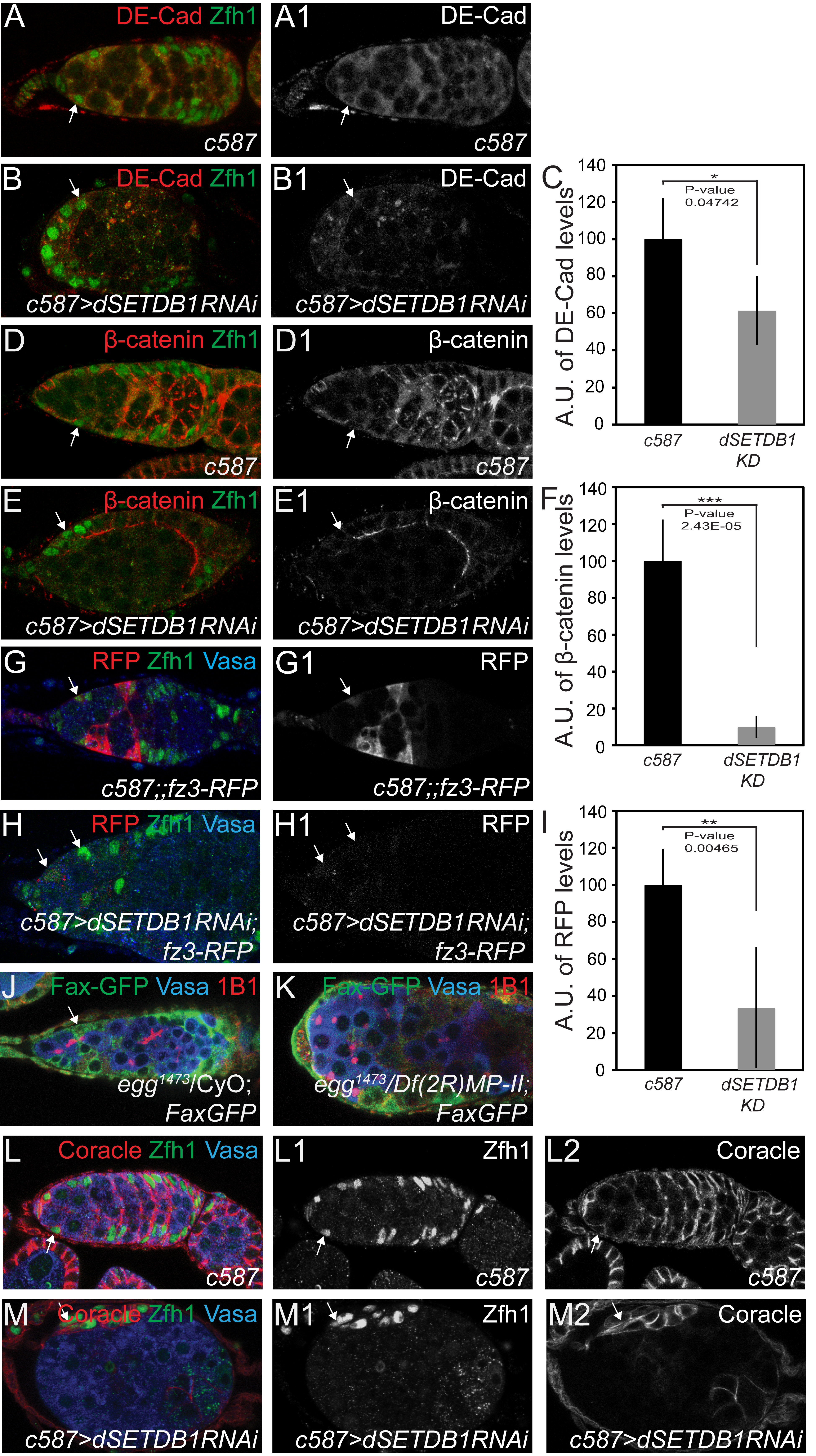

Supplement: S5 Fig — (A–B1) c587-GAL4 and escort cell knock down (KD) of dSETDB1 stained for DE-Cadherin (red), and Zfh1 (green) (white arrow) showing perturbed DE-Cadherin expression in dSETDB1 KD. (C) Quantification (n = 5) of DE-Cadherin levels in c587-GAL4 and dSETDB1 KD showing a significant difference in dSETDB1 KD. (D-E1) c587-GAL4 and dSETDB1 KD stained for β-catenin (red), and Zfh1 (green) (white arrow) showing perturbed β-catenin expression in dSETDB1 KD. (F) Quantification (n = 5) of β-catenin levels in c587-GAL4 and dSETDB1 KD showing a significant difference in dSETDB1 KD. (G-I) Control and dSETDB1 KD stained for RFP (red), Zfh1 (green) (white arrow) and Vasa (blue) showing perturbed RFP expression in dSETDB1 KD. (J-K) Control and dSETDBI mutant stained with GFP (green), Vasa (blue), and 1B1 (red) showing loss of encapsulation in dSETDB1 mutants. (L-M2) Control and dSETDB1 KD stained for Coracle (red), Zfh1 (green) (white arrow), and Vasa (blue) showing loss of encapsulation in dSETDB1 KD. (TIF) [file pgen.1005918.s005.tif]

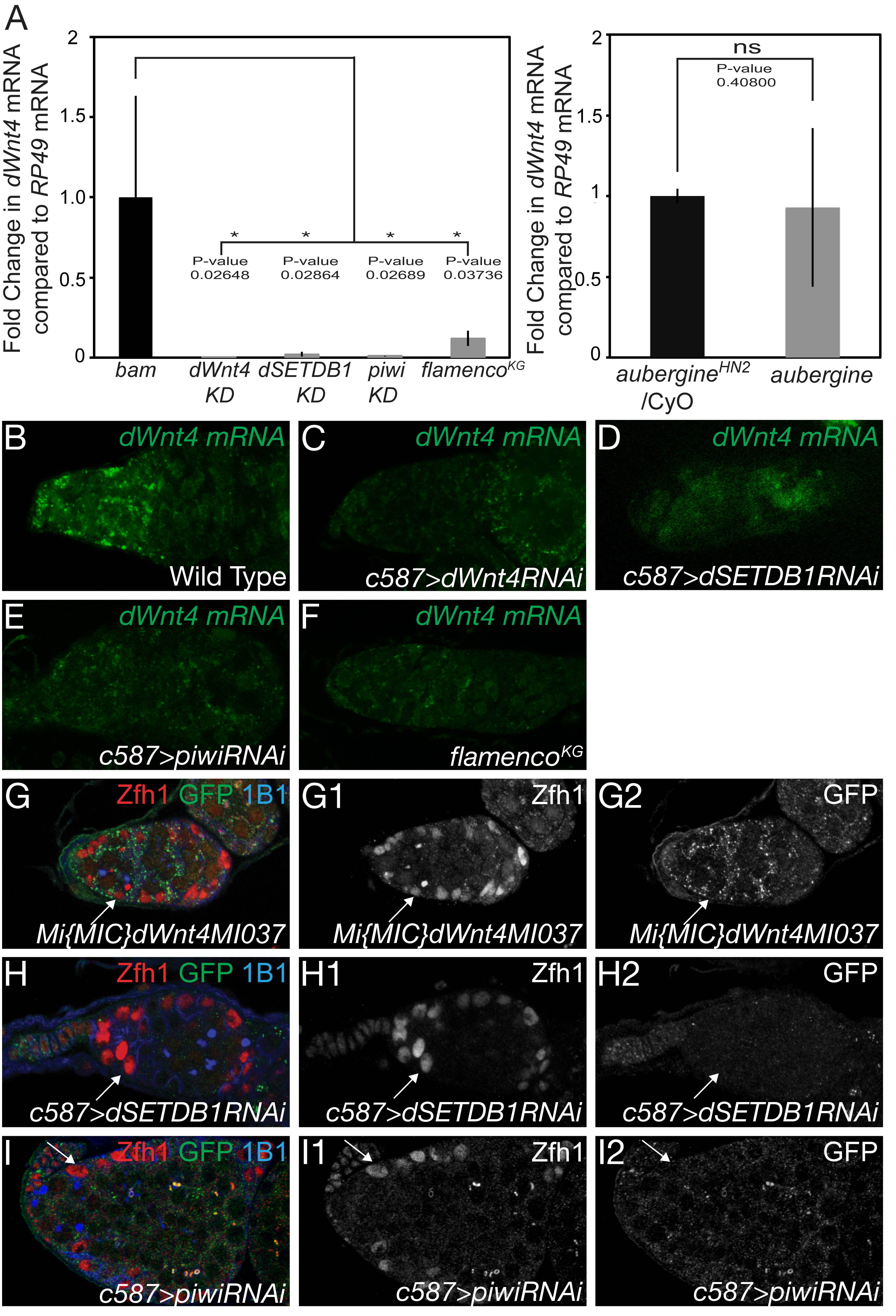

Supplement: S6 Fig — (A) qRT-PCR analysis showing a significant downregulation of dWnt4 mRNA levels compared to RP49 mRNA levels in escort cell specific knockdowns (KD) of dWnt4 KD, dSETDB1 KD, piwi KD and flamenco were compared to bam mutants. No significant change in dWnt4 mRNA levels was observed compared to RP49 mRNA levels was seen between aubergine mutants compared to its the heterozygous control. (B-F) Fluorescent in situ hybridization (FISH) for dWnt4 mRNA in wild type, dWnt4 KD, dSETDB1 KD, piwi KD and flamenco showing downregulation of dWnt4 in the soma compared to wild type. (G-G2) Germarium of a minos GFP (dWnt4 reporter) stained for Zfh1 (red), GFP (green) and 1B1 (blue) showing the expression of GFP primarily in the escort cells (white arrow). (H-I2) Germarium of dSETDB1 KD and piwi KD carrying dWnt4 reporter stained for Zfh1 (red), GFP (green) and 1B1 (blue) showing a downregulation of dWnt4 in the escort cells. (TIF) [file pgen.1005918.s006.tif]

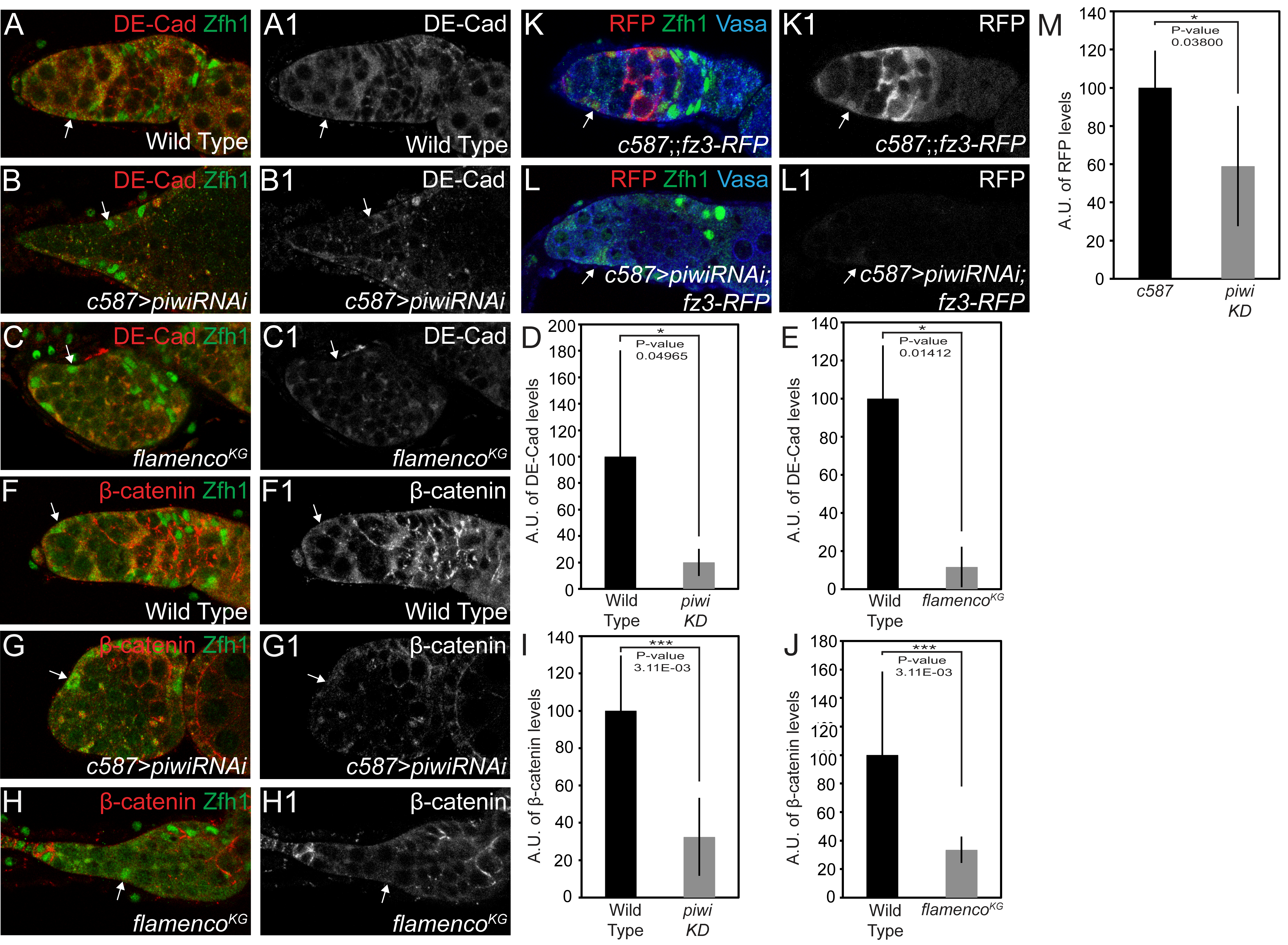

Supplement: S7 Fig — (A–C1) Wild type, escort cell knock down of piwi (piwi KD) and flamenco mutants respectively, stained for DE-Cadherin (red), and Zfh1 (green) (white arrow) showing perturbed DE-Cadherin expression in piwi KD and flamenco mutants. (D-E) Quantification (n = 5) of DE-Cadherin levels in wild type, piwi KD and flamenco mutants showing a significant decrease in mutants. (F–H1) Wild type, piwi KD and flamenco mutants stained for β-catenin (red), and Zfh1 (green) (white arrow) showing perturbed β-catenin expression in piwi KD and flamenco mutants. (I-J) Quantification (n = 5) of β-catenin levels in wild type, piwi KD and flamenco mutants showing a significant decrease in mutants. (K-K1) Germarium carrying a fz3-RFP transgene stained with RFP (red), Zfh1 (green) (white arrow) and Vasa (blue) showing expression of Fz3RFP in the escort cells. (L-L1) piwi KD carrying the same transgene stained with RFP (red), Zfh1 (green) (white arrow) and Vasa (blue) showing downregulation of Fz3 expression. (M) Quantification of RFP in the escort cells showing a significant downregulation in piwi KD. (TIF) [file pgen.1005918.s007.tif]

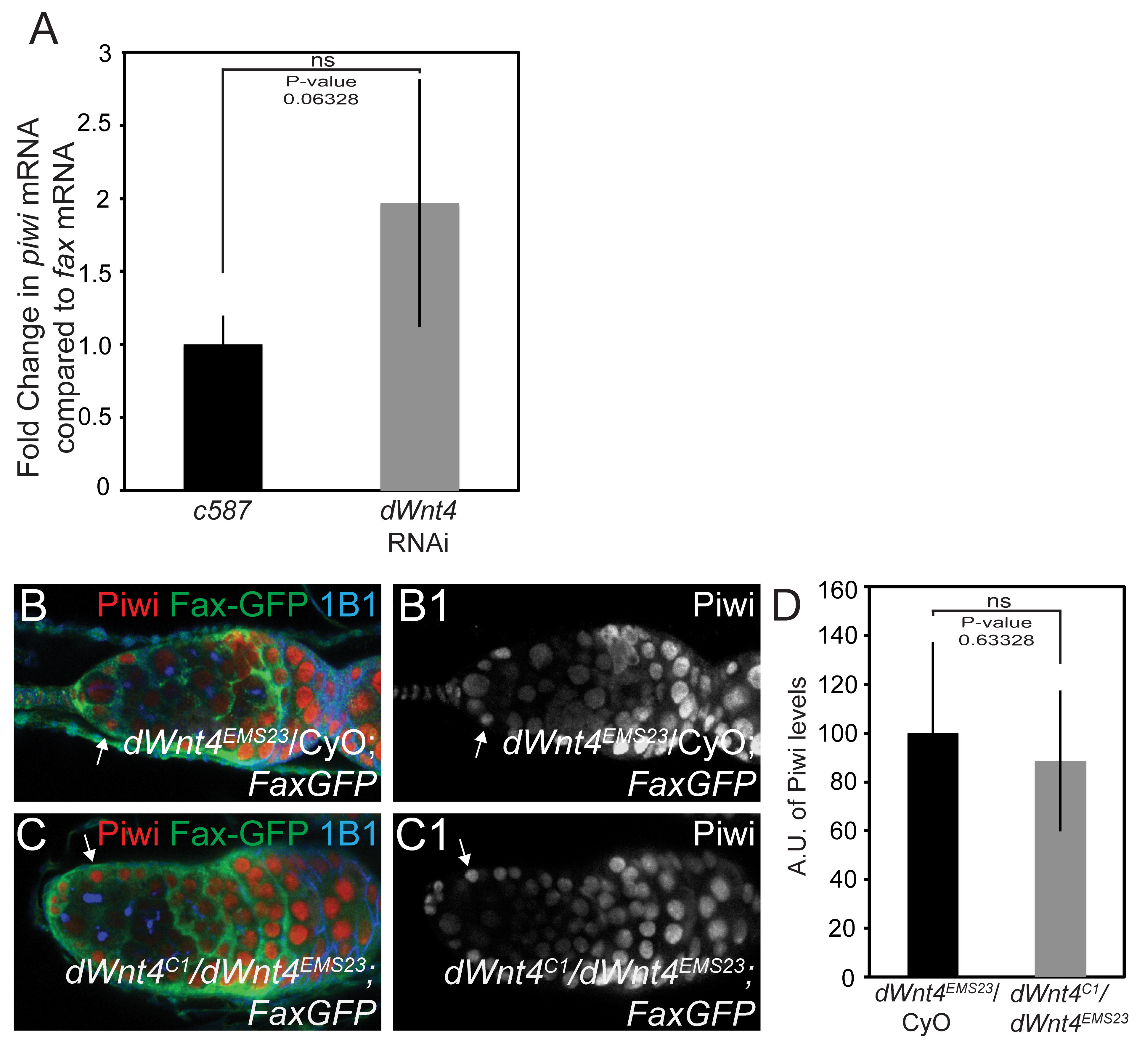

Supplement: S8 Fig — (A) qRT-PCR analysis showing no significant change in piwi mRNA levels between c587-GAL4 and germaria where dWnt4 has been specifically depleted in the escort cells. (B–C1) dWnt4 heterozygote and dWnt4 mutant fly stained for Piwi (red) (white arrows), GFP (green) and 1B1 (blue) showing similar Piwi expression in the escort cells. (D) Quantification (n = 6) of Piwi in escort cells showing a no difference in dWnt4 heterozygotes and dWnt4 mutants. (TIF) [file pgen.1005918.s008.tif]
